# Supplementary material for: Functional parcellation of mouse visual cortex using statistical techniques reveals response-dependent clustering of cortical processing areas
Source: PLoS Comput Biol. 2021 Feb 4;17(2):e1008548. doi: 10.1371/journal.pcbi.1008548 (PMC7888605; doi:10.1371/journal.pcbi.1008548)
Supplement: S1 Table — In the text, we analyzed Emx1-IRES and Nr5a1 Cre-lines from dataset 2 (Section 1.2.2). Here we present the number of neurons available from all the other Cre-lines that contain all the six visual areas considered in the paper. (PDF) [file pcbi.1008548.s008.pdf]

Cre-line	AL	LM	RL	AM	PM	V1
Slc17a7-IRES2 (Session A)	223	2184	97	138	1245	4499
Slc17a7-IRES2 (Session C2)	267	2140	70	149	1220	4232
Rorb-IRES2 (Session A)	794	767	975	516	511	1617
Rorb-IRES2 (Session C2)	292	421	1082	404	397	515
Cux2-CreERT2 (Session A)	2219	1820	1271	1060	1624	3558
Cux2-CreERT2 (Session C2)	497	358	1275	871	289	774
Rorb-IRES2 (Session A)	267	333	72	244	375	320
Rorb-IRES2 (Session C2)	109	194	68	237	141	242
