# Supplementary material for: Functional parcellation of mouse visual cortex using statistical techniques reveals response-dependent clustering of cortical processing areas
Source: PLoS Comput Biol. 2021 Feb 4;17(2):e1008548. doi: 10.1371/journal.pcbi.1008548 (PMC7888605; doi:10.1371/journal.pcbi.1008548)
Supplement: S2 Table — In the text, we presented the classification accuracy for Emx1-IRES and Nr5a1 Cre-lines from dataset 2 (Table 4). Here we present the same for all the other Cre-lines in dataset 2 that contain all the six visual areas considered in the paper. (PDF) [file pcbi.1008548.s009.pdf]

Cre-line (Session)	Stimuli	Accuracy of Supervised Classifier			
		GMM	SVM	ANN	Bayes
Slc17a7-IRES2 (Session A)	Natural Movie 1	58.0 (±1.28)	65.4 (±0.49)	65.0 (±0.577)	57.10 (±0.98)
	Natural Movie 3	68.6 (±1.44)	71.0 (±0.26)	71.4 (±0.13)	66.9 (±0.82)
	Resting State	55.9 (±1.13)	61.2 (±0.85)	59.9 (±1.08)	44.6 (±1.68)
Slc17a7-IRES2 (Session C2)	Natural Movie 2	59.0 (±1.01)	63.0 (±0.33)	62.8 (±0.50)	55.7 (±0.59)
Rorb-IRES2 (Session A)	Natural Movie 1	42.2 (±1.44)	45.2 (±0.68)	44.9 (±0.78)	42.0 (±0.47)
	Natural Movie 3	48.5 (±1.77)	53.0 (±0.30)	53.4 (±0.57)	48.7 (±0.84)
	Resting State	65.3 (±1.62)	67.8 (±1.05)	68.4 (±1.64)	63.5 (±1.80)
Rorb-IRES2 (Session C2)	Natural Movie 2	46.8 (±0.59)	48.3 (±0.78)	47.6 (±0.57)	44.6 (±1.31)
Cux2-CreERT2 (Session A)	Natural Movie 1	37.5 (±1.16)	43.0 (±0.68)	43.5 (±0.70)	39.3 (±0.72)
	Natural Movie 3	39.0 (±0.82)	47.7 (±1.27)	50.1 (±0.93)	38.7 (±1.09)
	Resting State	45.8 (±1.15)	48.6 (±0.98)	47.3 (±1.005)	39.2 (±0.69)
Cux2-CreERT2 (Session C2)	Natural Movie 2	45.6 (±1.47)	46.9 (±1.66)	46.4 (±1.70)	44.9 (±1.30)
Rbp4 (Session A)	Natural Movie 1	34.6 (±2.20)	34.6 (±1.88)	34.5 (±2.17)	33.1 (±2.00)
	Natural Movie 3	38.1 (±2.50)	39.7 (±2.23)	38.2 (±2.31)	35.9 (±2.27)
	Resting State	35.6 (±1.79)	41.0 (±2.73)	39.9 (±2.92)	33.7 (±2.75)
Rbp4 (Session C2)	Natural Movie 2	34.0 (±1.64)	34.2 (±1.09)	32.5 (±1.51)	31.4 (±0.81)
