# Supplementary material for: Functional parcellation of mouse visual cortex using statistical techniques reveals response-dependent clustering of cortical processing areas
Source: PLoS Comput Biol. 2021 Feb 4;17(2):e1008548. doi: 10.1371/journal.pcbi.1008548 (PMC7888605; doi:10.1371/journal.pcbi.1008548)
Supplement: Attachment — Submitted filename: response_to_reviewers.pdf [file pcbi.1008548.s010.pdf]

Response to reviewers

"Functional Parcellation of Mouse Visual Cortex Using Statistical Techniques Reveals Response-Dependent Clustering of Cortical Processing Areas" (PCOMPBIOL-D-20-00156)

We thank the three reviewers for their detailed comments. To address these comments, we have carried out significant additional analyses and made extensive changes to the manuscript. Our changes include 4 new text figures (Figures 4, 7, 8, and 9), 2 new text tables (Tables 2 and 4), 4 new supplementary figures (Figures S1, S4, S5, and S6) and 2 new supplementary tables (Table S2, S3). A point-by-point response is provided below.

Reviewer 1

Reviewer point 1.1 — *"Kumar et al. studied wide-field GCaMP signals in 6 cortical visual areas in the mouse. Pixels clustered into 6 groups with boundaries that match retinotopic borders, indicating that each visual area is distinct. Unfortunately, what causes these areas to cluster is unknown. With no information on the basis of clustering it's difficult to assign significance to the clusters. Furthermore, the distinguishing feature is not related to visual stimuli since the same clusters were derived from spontaneous activity, suggesting that the clusters contain no information about the roles of these areas in processing visual information. In short, the paper reports an observation – that pixels cluster – but doesn't explain why. The observation would appear to have little significance." (See also Reviewer 3, point 7)*

Response 1.1: We appreciate this important comment. We have carried out two new analyses to provide additional insight into the visually driven and resting state response patterns that enable the classification. First, we have computed the average intra-area and inter-area correlations between the neurons/pixels, for wide-field and two-photon datasets. Fig R1 **A**, **B** shows the correlation computed for raw wide-field data using natural movies and resting state responses, respectively. Similarly, Fig R1 **E**, **F** shows the same result for raw data from the Emx1-IRES subset of the two-photon dataset. For both datasets, the average intra-area correlation is consistently higher than the inter-area correlation. Even with resting state responses, in the absence of overt visual stimuli, the responses are more correlated within an area. The ratio of average intra- to inter-area correlations calculated on raw responses were 1.1 and 2.5 for the wide-field and two-photon datasets, respectively. The raw signals were preprocessed using PCA and LDA before they were given to the classifiers. In Figs R1 **C**, **D**, **G**, **H**, the correlations computed in the LDA domain are shown. The LDA space significantly improved the ratio of average intra-area and inter-area correlations to 8.8 and 9.7 for the wide-field and two-photon datasets, respectively. In Fig R2, we show the 2D visualization of LDA subspace obtained using wide-field (mouse M4) and two-photon (Emx1-IRES Cre-line) datasets. T-distributed stochastic neighbor embedding (tSNE, Maaten and Hinton [2008]) was used to convert the multi-dimensional LDA subspace into a visualizable 2D space. The LDA subspace is able to cluster neurons from different areas owing to correlated examples from the training data, in both visually driven and resting state responses from wide-field and two-photon datasets (Fig R2). By applying different supervised classifiers to these subspaces, we were thus able to identify the area labels with high accuracy.

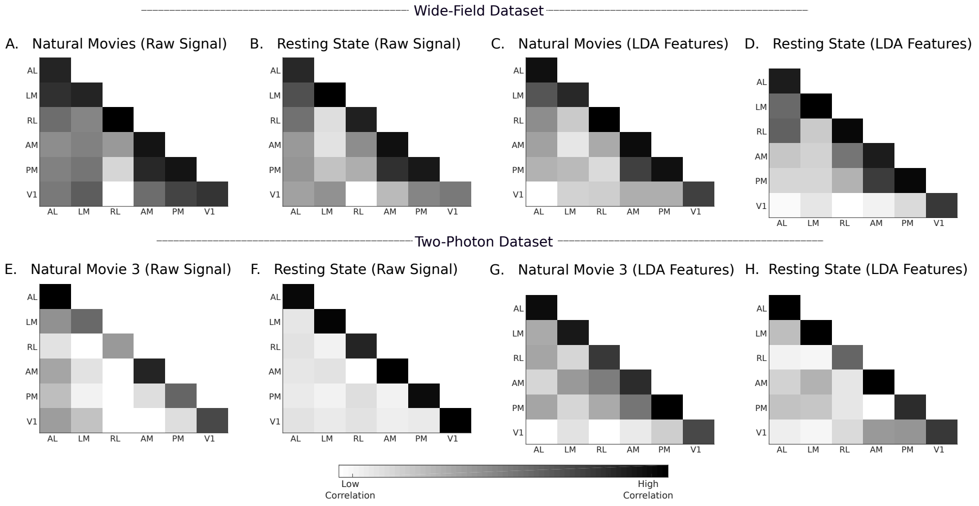

Fig R1. Intra-area and inter-area correlations computed on raw responses and LDA features. A-D) Correlations computed from mouse M4 of wide-field dataset. **E-H**) Correlations computed from Emx1-IRES Cre-line of two-photon dataset. The correlations are computed as averages over all unique pairs of neurons/pixels in the test data, which were not used to train the LDA projection matrix. In Supporting Information Figs S5 and S6, we show the correlations among raw responses and LDA features for all the other animals and data.

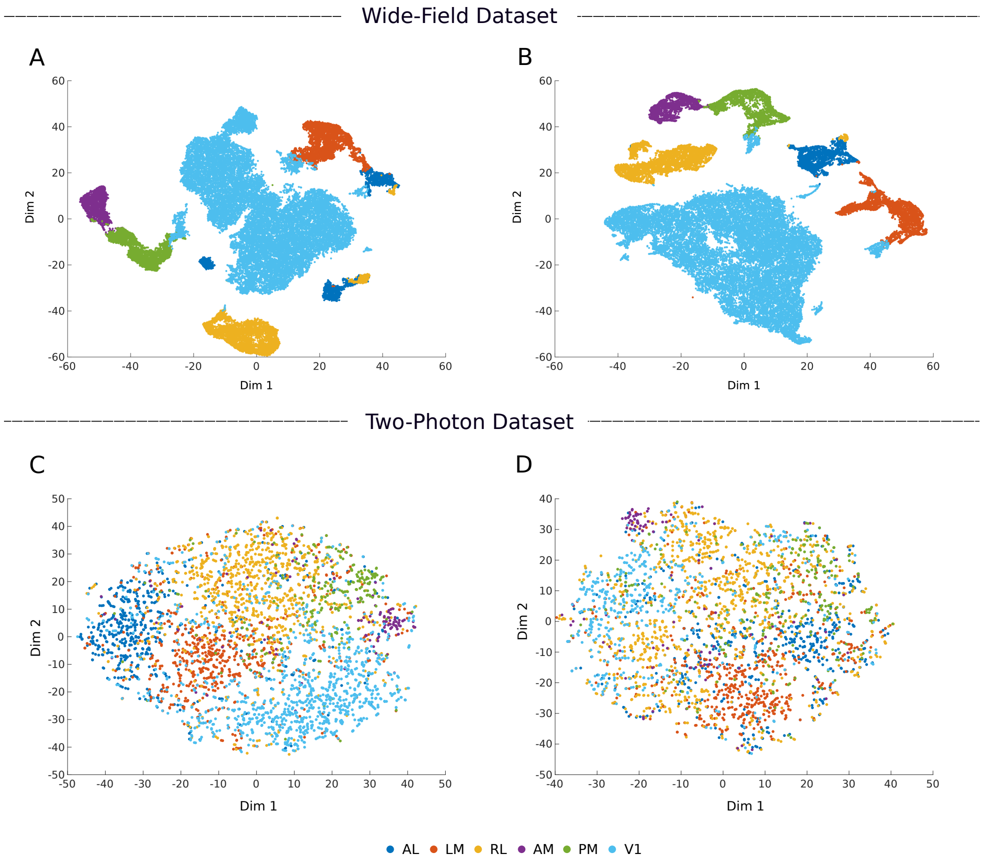

Fig R2. Two-dimensional representation of the supervised LDA subspace. A, B) LDA subspace of wide-field dataset (mouse M4). **C, D)** LDA subspace of two-photon dataset (Cre-line Emx1-IRES). The plots on the left (**A**, **C**) are obtained from natural movie responses and that on the right (**B**, **D**) are obtained from resting state responses.

Both PCA and LDA are simple linear projections of the data. An illustration of LDA projection is shown in Fig R3. The LDA projection (in Fig R3 **B**) is able to minimize the within-class variability and increase the between-class distance because the clusters are distinct in the input x, y coordinate space (Fig R3 **A**). Thus, we argue that the LDA space is able to cluster neurons from different areas (Fig R2) using examples from the training data owing to the stronger intra-area correlations found in raw signals. Figs R1 and R2 are added as Figs 8 and 9 in the revised manuscript, and the correlation analysis has been added to the **Discussion** (Section 3) in the paper.

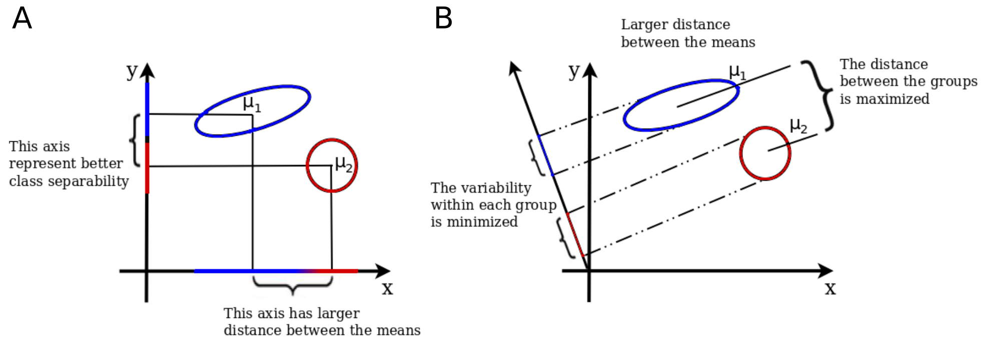

Fig R3. Illustration of LDA. A) Two cluster in x, y coordinate space. B) A possible LDA projection that minimizes within class variability while increasing between class distance. (Redrawn from Elhabian and Farag [2009])

In a second new analysis, we have analyzed resting state and visually driven responses in more detail, using different response durations and additional data on intrinsic activity from the Allen Institute dataset. We show that retinotopic area classification is better with stimulus-driven responses than resting state responses of the same duration, particularly when multiple trials are averaged. This has been added to **Results** (Section 2.3) and new Fig 7 in the text.

These findings demonstrate two important features of visual areas in mice, relevant to processing of visual stimuli. First, they are consistent with the fact that each cortical area is characterized by a unique pattern of internal connections and circuits. Some of these may be common to many areas of cortex (eg., local recurrent excitatory connections) whereas others are crafted by a combination of specificity and plasticity (eg., local inhibitory connections, long-range

excitatory connections). These connection patterns come into play even with internally generated activity which characterizes resting state responses. Thus, the intra-areal correlations are higher than inter-areal correlations for both visual and resting state responses. Second, visual stimuli are stronger drivers of internal circuits than resting state activity. Thus, the visually driven responses have higher classifier accuracy than resting state responses for given response durations, and in some instances the resting state responses never reach the accuracy of visual responses (Fig 7). This has been added to the **Discussion** (Section 3), and also to the **Abstract**.

Reviewer point 1.2 — *The paper's littered with sentences that appear disconnected from the results and conclusions. I'll give two examples. The second sentence of the abstract reads: 'The extent to which these areas represent discrete processing regions remains unclear.' The paper brings no clarity. Why suggest the study is of visual processing? And in the results 'These results indicate that each visual area has a characteristic signature that is represented in the responses to visual stimuli presented and can be revealed with a variety of visual stimuli.' What signature? Information on this signature is conspicuously missing. And about the only thing we know is that the signature's not related to presented visual stimuli. This sentence is comprehensively at odds with the results. The text frequently suggests the paper will be about the different visual stimuli that drive these visual areas, but there's no information here on this topic.*

My sense is that the study didn't lead in the direction the authors had expected. If so, it would be best to let go of the intended direction of the project and address what they can with the results. In particular, the aim and conclusions need to be clear and clearly related to the results.

Response 1.2: We now clarify that the paper addresses the question of whether six retinotopic visual areas of the mouse cortex can be classified based on their activity patterns in response to different visual stimuli or in the resting state. We have rewritten the **Abstract** to emphasize the motivation and our findings, clarified the goals of our work in the **Introduction**, and modified the **Conclusion**. We have modified the second sentence in the **Abstract**, and removed references to 'response signatures' in the **Abstract** and **Results**. The above statement in Results has been changed to: "These results thus indicate that the responses of different visual areas to a range of visual stimuli can be used to reliably and accurately classify their borders".

Our paper addresses the question we asked and gives clear answers, and this has been laid out in the revised **Abstract** and **Results**. First, we show that areas can be classified based on visually driven activity. Second, we show that resting state responses are an important complement to visual responses in classifying areas. Visual as well as resting state responses do not arise de novo; rather they both reflect specific underlying input-output connections and circuits in each area. These connections can be activated by internally generated activity without overt visual stimuli, or explicitly by visual stimuli. In the revised manuscript, we now also show resting state results for the two-photon Allen Institute dataset (detailed in Response to Reviewer 2, point 2). Thus, we now show comparable results from two different datasets each with multiple stimuli along with resting state responses, demonstrating that resting state and visual responses can be used to classify area borders. We also provide new analyses that intra-areal responses are more correlated than inter-areal responses, for both visually driven and resting state activity, which we hypothesize drives classifier accuracy (See Response 1.1). This has been added to the **Discussion** (Section 2.3) in the paper.

Our analysis does not speak further to what features of neuronal responses are responsible for the classification. The analysis pipeline does not use any explicit configuration of stimuli except averaging the response across trials. Hence, we have removed mention of response signatures from the text and modified the title of the paper.

Reviewer point 1.3— *What is the genotype of the transgenic GCaMP mice? Also, the breeding scheme.*

Response 1.3: The mice were generated by crossing Ai93 (TITLa-tTA) with Emx1-IRES-Cre mice lines from Jackson Labs. This is now mentioned in **Methods** (Section 1.1.1)

Reviewer point 1.4 — *What's the eye-to-screen distance and visual angle subtended by the monitor.*

Response 1.4: The eye-to-screen distance was 12 cm. Dimension of the monitor was 52.7×33.6 cm, subtending a visual angle of $131° \times 108°$ at that distance. This has been added to **Methods** (Section 1.1.1)

Reviewer point 1.5 — *What was the size (in degrees) of the visual stimuli?*

Response 1.5: The visual stimuli for retinotopic mapping was a narrow bar as described in the paper. The length of the bar was the full length across the display; depending on the orientation of the bar, it was either $131°$ at horizontal orientation or $108°$ at vertical orientation. The width of the bar was kept at $14°$ for either orientation. This has been added to **Methods** (Section 1.1.1). Full-field visual stimuli (gratings, movies) were presented on the entire monitor.

Reviewer point 1.6 — *Figure 1D. Why does the field sign map appear so patchy? It's different from the maps produced by others with wide-field GCaMP imaging. See for example Zhuang et al. eLife 2017. I would guess perhaps the SNR of the retinotopic maps are poor?*

The boundaries of the 6 core visual areas were defined according to criteria described in [9]' This statement is conspicuously untrue. If the authors intended to replicate the field sign mapping technique of Garrett et al., they have failed. In Garrett et al. the borders are, by definition, where the field sign crosses zero. In figure 1D, the borders are not at zero. Many, perhaps all the borders are at some negative field sign value. The authors need to take a closer look at their code. They also need to provide a more detailed explanation of their mapping procedure and, ideally, the code.

Response 1.6: Our procedure is identical to Garrett et al. [2014]. According to Garrett et al. [2014], the visual area boundaries were defined by morphological post-processing over the sign maps. Our sign maps look similar to Garrett et al. [2014] (eg., see their Fig 2), and they are no more patchy than any other study that has described such maps (eg., S1 Fig. in Waters et al. [2019]). Some studies that impose a 'standard' map on the cortex provide little actual mapping data and give the impression that maps are smoother than they actually are on close examination (eg., Andermann et al. [2011]). Importantly, in our study we take into account the natural variability of areal borders in each mouse to build classifiers effectively. In the revised manuscript, Fig 1D shows the visual field sign for each area to avoid confusion. In Fig R4, we show the azimuth and altitude contours along with the derived borders for all the mice used in the paper. The visual field representation is the ground truth for each area, and is similar across mice and also to other studies which have shown such maps (Garrett et al. [2014]; Zhuang et al. [2017]). Fig R4 is added as Supporting Information Fig S1.

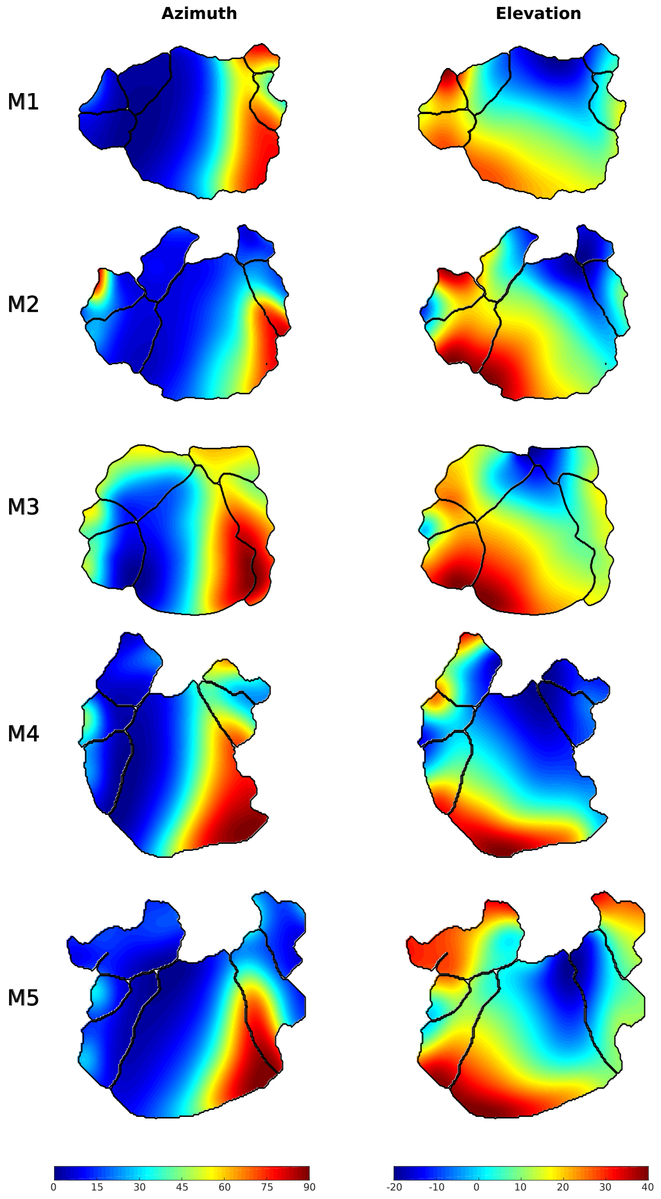

Fig R4. Horizontal and vertical retinotopy within 6 visual areas of all the mice used in the paper. Cortical areas of the left hemisphere are shown. Azimuth 0° and 90° correspond to the midline and the far periphery of the contralateral visual field, respectively. Negative values of elevation represent lower visual field and positive values represent upper visual field.

Reviewer point 1.7— *Table 1 provides incomplete information on the stimuli. For 1, give the SF and TF. For 2, the TF and direction. For 3, the SF and direction. We also need luminance, contrast and size of the stimulus.*

Response 1.7: The missing information is shown in Table R1 (given as Table 1 in the main paper). The table has been updated.

Table R1: **Summary of different stimuli shown to mice**

S. No	Stimuli Name	Description
1	Directions/ Orientation	16 different sinusoidal gratings with varying direction from 0° to 360° with a step of 22.5^o. The spatial and temporal frequencies were fixed at 0.03 $_{\text{cycles/degree}}$ and 3 Hz, respectively. Michelson contrast of 0.8 was used.
2	Spatial-Frequency	5 different sinusoidal gratings with spatial frequency increasing exponentially from 0.01 $_{\text{cycles/degree}}$ to 0.16 $_{\text{cycles/degree}}$. The temporal frequency was fixed at 3 Hz. For each spatial frequency, the direction was varied from 0° to 360° with a step of 45°. Michelson contrast of 0.8 was used.
3	Temporal-Frequency	5 different sinusoidal gratings with temporal frequency increasing exponentially from 0.5 Hz to 8 Hz. The spatial frequency fixed at 0.03 $_{\text{cycles/degree}}$. For each temporal frequency, the direction was varied from 0° to 360° with a step of 45°. Michelson contrast of 0.8 was used.
4	Natural Movies	4 different movies with natural scenes. For each movie additional noisy versions were created by perturbing their spatial correlations, as demonstrated in Rikhye and Sur [2015].

Reviewer point 1.8 — *2.1 The supervised classifiers are described in numerical detail, but I gained no insight into the differences between the classifiers. Why these classifiers? Was there reason to use several?*

Response 1.8: We have used different classifiers to show that this result is not based on a single classifier. We have shown that the proposed methods work with generative (GMM, Unimodal Bayes) and discriminative (SVM, ANN) classifiers. We have also shown the approach to work with linear (Unimodal Bayes, SVM) and non-linear (ANN, GMM) classifiers. This shows that the obtained results are mainly because of the proposed PCA and LDA subspace rather than the classifier. In addition, the fact that a non-linear classifier performs similarly as linear one suggests that major difference between areas has been captured by linear models. This has been added to the **Discussion** (Section 3).

Reviewer point 1.9 — *Figure 4D and E. The LM and RL labels are swapped.*

Response 1.9: The labels have now been fixed in Fig 4D

Reviewer point 1.10 — *Figure 5 and later figures. Why are the maps broken down by visual stimulus when earlier results were not. And why break them down by visual stimulus when clustering needs no visual stimulus?*

Response 1.10: This is a misunderstanding. For all the classifiers (from Fig 3), the results are broken down by the different visual stimuli and mice. We show that we are able to classify/cluster the areas using all the visual stimuli or resting state with no overt stimuli. Further, in Section 2.3, we also show that classification is better with stimulus-driven responses than resting state responses.

Reviewer point 1.11 — *2.3 Resting vs stimulus induced response. I failed to grasp the aim of this section.*

Response 1.11: Specific activity patterns including reverberation-like activities during resting states (absence of overt visual stimuli) in visual cortex have been observed by several labs, even in anesthetized animals (see Yao et al. [2007]). Given these observations, the logic behind our experiment was to use our proposed methods to further test whether spontaneous activities encode any area-specific information. We were not able to detect any significant reverberations, most probably due to the slow dynamics of calcium signals. Nevertheless, we found that resting state activity could be used to discriminate visual areas. This is a novel finding, and we wished to examine it further, which we have done in section 2.3.

In Sections 2.1 and 2.2, the natural movies, and other stimuli were presented multiple times and averaged to obtain stimulus-induced responses. Since a structured trial cannot be defined for resting state responses, the dF/F of the signal was used as the input. In section 2.3, we now compare resting state responses with single-trial and trial-averaged stimulus induced responses by fixing the response duration. This analysis enables an in-depth analysis of the results obtained with and without stimuli: we show that stimulus-driven responses contain better discriminating responses than the resting state when response lengths are limited to 100 secs (see also Response 3.8).

We have revised Section 2.3 and added new Fig 7 (replacing previous Figure 6).

Reviewer point 1.12 — *The unsupervised clustering simply fails. Is there a reason to include it? The stated conclusions are weak at best. (See also Reviewer 2, point 3 and Reviewer 3, point 3)*

Response 1.12: Following suggestions from Reviewers 2 and 3 as well, we performed a new set of experiments to examine in more detail the unsupervised clustering analysis. Below we summarize our experiments and results:

Experiments:

- We introduced new metrics to measure the effectiveness of the clustering result. V-measure (Rosenberg and Hirschberg [2007]), was used as a metric to compare the clusters obtained by our approach and the areas defined by retinotopy. V-measure is computed as the harmonic mean of completeness and homogeneity of the obtained clusters.
- For a control experiment the initial clusters were spatially shuffled, and the clustering was repeated.
- In addition to BIC, we tried a new merging criterion which measures the generalized spread in the cluster by computing the determinant of its covariance matrix. The results of old and new approaches are given in Figs R9 and R10, respectively.

Results:

- The new results obtained using determinant of the covariance matrix as the merging criteria (Fig R10) gave slightly better results than the previous method using BIC (Fig R9). The determinant of the covariance matrix scored an average V-measure of 0.37 while the clustering using BIC scored a V-measure of 0.30.
- Shuffling the initial clusters randomly gave an average V-measure of 0.06 and 0.09 for BIC and determinant based clustering, respectively.

However, the results obtained in Figs R9 and R10 are still very different from the retinotopically defined areas. Since it was hard to interpret these clusters, we decided to keep this analysis just as a part of Response to Reviewers and have removed the analysis from the paper (as recommended by reviewers 1 and 3). In the main article we now mention that unsupervised clustering was attempted; however, we were not able to cluster them meaningfully (**Discussion**, Section 3). Appendix 1 in this response details our additional experiments on the unsupervised approach.

Reviewer 2

Reviewer point 2.1 — *This paper addresses the question of whether the retinotopic visual cortical areas of the mouse can be discovered from their activity patterns in response to visual stimuli or in the resting state. The study concludes that retinotopically defined areas have unique activity profiles that allows their identification based on supervised and semi-supervised methods. However, unsupervised approaches fail to recover these areas with great accuracy, suggesting that despite differences between areas, there is also a great deal of overlap between areas. The question posed is an interesting one, and overall the results are convincing. I like the general approach and think this approach will be valuable for many future questions, even beyond studies of visual cortex. I therefore support publication of this work if the points below can be addressed.*

Response 2.1: We appreciate the reviewer's view of the paper. Below we have addressed reviewer 2's comments in detail.

Reviewer point 2.2 — *In the discussion and conclusions, a lot of emphasis is placed on the resting state data. The authors emphasize that some of the separation between areas could be due to intrinsic activity rather than visual responses. However, there are only two mice for the resting state data, which seems like too small of a sample size. Either these claims should be lessened or more resting state data should be added.*

Response 2.2: In the revised manuscript, we have added the analysis of intrinsic activity from the Allen Institute dataset. We show that the supervised classifier trained using resting state activity from this dataset also performs very similar to the trial-averaged natural movie responses. These observations include two Cre-lines in the main article (Table 4) and four other Cre-lines in Table R4. Table R4 is included as Supporting Information Table S3.

Reviewer point 2.3 — *I like the analysis of shuffling the area labels in the supervised analysis to show the chance level. I think similar analyses would be nice for the other parts of the paper too. For example, for the unsupervised clustering, it might be interesting to compare clustering metrics for the real data and data in which the pixel locations are shuffled. This could provide some measure of how much structure can be discovered in the real data relative to what would emerge from random data. In general these comparisons are helpful to provide the reader with a bound on what can be expected by chance. (See also Reviewer 1, point 12 and Reviewer 3, point 3)*

Response 2.3: As suggested by the reviewer, we carried our further analysis of unsupervised clustering. Our experiments and results are summarized in Response 1.12, and described in Appendix 1. We found little improvement, and have removed the analysis from the paper (as recommended by Reviewer 1, point 12 and Reviewer 3, point 3).

Reviewer point 2.4 — *My understanding is that the unsupervised analysis was only performed on the widefield calcium imaging data. It was a bit hard to figure this out in the text, so I apologize if I am incorrect. If my statement is correct, then it would be nice in addition to see the unsupervised analysis on the single cell data. The single cell data lack spatial correlations that are present in the widefield data, as the authors note. It would be interesting to see if similar clusters could be uncovered with the single cell data.*

Response 2.4: We have not tried unsupervised clustering of the two-photon dataset. For clustering the wide-field dataset, we merged only clusters that are spatially neighbors. Furthermore, we use grids of size 25 to 49 pixel as starting points. In the two-photon dataset, without the spatial information the initial grids and the merging criteria need to be changed completely. Without the spatial relation of pixels/neurons, the problem is even more under-constrained.

Moreover, as stated in Response 1.12, Response 2.3, and Appendix 1, since it was hard to interpret clusters obtained from the unsupervised approach, we decided to keep this analysis just as a part of Response to Reviewers and removed it from the main paper. In the main article we discuss (in Section 3) that unsupervised clustering was attempted, but that we were not able to cluster the areas meaningfully.

Reviewer point 2.5 — *The tables of accuracies for the supervised and semi-supervised analyses are nice, but it would also be interesting to see the confusion matrices for these analyses. It would be interesting to some readers to see which areas are more similar to one another and thus get confused with one another more frequently. Such a confusion matrix could support some of the claims about lateral versus medial differences that the authors make using the unsupervised analysis. (See also Reviewer 3, point 5)*

Response 2.5: In Fig R5, we show examples of confusion matrices, obtained using mouse M1 from wide-field dataset and Emx1-IRES Cre-line from two-photon dataset, respectively. In Supporting Information Fig S4, we show the same for the entire dataset. For the wide-field dataset, responses from other areas were mostly predicted as V1 (Fig R5), which is not unexpected since V1 projects to each of the other areas. The confusion observed in the two-photon dataset were variable. Importantly, however, for both datasets, the majority of neurons were predicted correctly. This has been added to the **Results** in Section 2.1. Fig R5 is included as a new figure (Fig 4) in the text.

Reviewer point 2.6 — *The raw retinotopic map data for all mice should be shown in addition to the post-processed boundaries. This is important to evaluate the quality of the input to parcellate the areas into retinotopic divisions. In particular I ask about this because I was surprised by how much variance there was in the size and location of the areas. For example, in some mice AM is anterior and medial to PM, whereas in other mice AM is anterior and lateral to PM. Also, sometimes AM is directly bordering V1, and other times it is not. I was surprised by the location of AM in Figure 1D. Typically AM is adjacent to V1. Similar variance is seen for other areas. I am not sure it matters greatly for this study, but I have some concern that the area labels may be inaccurate in some mice, such as the case for AM that is not adjacent to V1 (Figure 1D). (See also Reviewer 1, point 6 and Reviewer 3, point 2)*

Response 2.6: A recent study (Waters et al. [2019]) explored the variability of the different visual areas in 60 mice. A previous study (Garrett et al. [2014]) also shows areas from multiple mice. There are significant variations in the

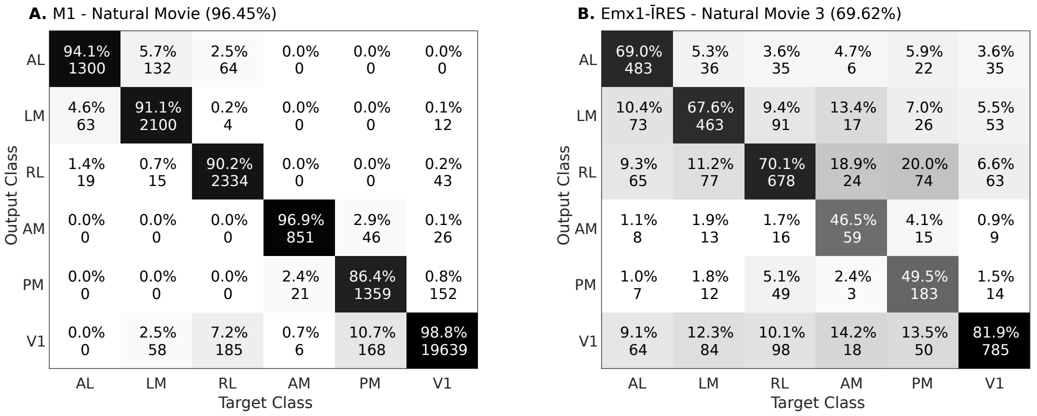

Fig R5. Confusion matrices for test data obtained using supervised classifier. The diagonal values denote the precision (in %) of each class. Off-diagonal values denotes the false prediction rate (in %) for the predicted class given the actual class. **A**) Confusion matrix obtained using responses of Mouse M1 and Natural Movie stimuli. **B**) Confusion matrix obtained using the Cre-line Emx1-IRES and Natural Movie 3 stimuli from dataset 2. In Supporting Information Fig S4, we show the confusion matrices for all the remaining data.

size, shape and location of each of the 5 visual areas that are close to V1. As the reviewer requested, in Fig R4, we show the azimuth and altitude contours along with the derived borders for all the mice used in the paper. Fig R4 has been added as Supporting Information Fig S1.

Reviewer point 2.7 — *Currently all the analyses are done within a mouse, which is sensible. However, I was wondering if the authors tried across mouse analyses. For the supervised analyses, what do the results look like if the classifiers are trained on mouse 1 and tested on mouse 2? For the unsupervised analysis, is there a way to see if the clusters identified in mouse 1 then provide predictive power for mouse 2? Across mouse analysis might further support claims of structure made by the authors. (See also Reviewer 3, point 15)*

Response 2.7: We tried pooling data from mice M1 and M2. When training data was sampled from this pooled dataset, the classifier worked with accuracy of about 90% for test data, from both M1 and M2. However, when trained on M1 and tested on M2, the result did not scale. This shows that each mouse's data is quite variable, and the proposed classifier only works for the mice that were seen during training. Indeed, it would be very exciting to train a model that could be generalized across mice, as this would tease apart the covarying factors from invariant factors in the area-area relationships. However, it is not clear whether such invariant relationships exist at this level. Further studies are needed to address this important question.

Reviewer point 2.8 — *What were the mice doing during the imaging experiments? Were they moving? Could movement contribute to the results? Recent studies have emphasized the importance of movement to visual cortical activity (see PMIDs: 20188652, 31551604, 31000656).*

Response 2.8: The mice were restrained in a narrow tube and thus did not have large movements during the wide-field imaging experiments. In addition, all analysis was based on trial averaged data. Thus, random small movements would not contribute. We have added this in **Methods**, Section 1.1.1. The Allen Institute dataset that we analyzed has responses recorded while the mice were allowed to be free-running and thus in many instances were not stationary.

Reviewer point 2.9 — *Some references to recent papers using related methods were missing and should be added. PMIDs: 32282806, 30772081.*

Response 2.9: PMID 32282806 used Local NMF and PMID 30772081 used CNNs to derive insights from wide-field responses of mouse visual cortex. Citations to these papers have been included in **Introduction**.

Reviewer 3

Reviewer point 3.1 — *The authors use widefield and 2-photon imaging data from the mouse visual cortex to train classifiers to identify the different visual areas. They find that supervised and semi-supervised classifiers perform well, identifying pixels or neurons with high accuracy. The fact that they do so using even just the neural*

responses to one 4.5 second movie is remarkable. The authors go on to show that unsupervised classifiers do not identify the different visual areas with high accuracy, but do capture some of the functional organization of the visual cortex. These results indicate that there are distinct physiological profiles for the different visual areas – or from the unsupervised results at least from groups of visual areas. I found the work to be interesting, and the paper did a great job of explaining the different techniques and conclusions. I do have some concerns, that I hope are reasonably addressable.

Response 3.1: We appreciate the reviewer's view of the paper. Below we have addressed reviewer 3's concerns point by point.

Reviewer point 3.2 — *The retinotopic maps shown in these figures are somewhat different from the retinotopic maps I see in the literature (namely Zhuang et al 2017 and Garrett et al 2014) – specifically in regards to the location and borders of RL. In the two papers mentioned above, RL sits at the top of V1, in contact with both AL and AM on either side. I understand that this area has difficult retinotopy, however, it seems possible that the pixels at the top of V1 are being mis-assigned to V1 and should really be within RL – which could had different effects on the different supervised/semi-supervised/un-supervised results. I encourage the authors to look more closely at the assignment for RL, or perhaps to consider excluding it from these analyses (or weighting the accuracy for that area differently). (See also Reviewer 1, point 6 and Reviewer 2, point 6)*

Response 3.2: See Response 2.6. We refer to a recent study (Waters et al. [2019], which added to data shown in Zhuang et al. [2017]) as well as a previous one (Garrett et al. [2014]) in which variability in location and size of different visual areas were described. In a few of the cases, RL and AL do not share a border or are not adjacent to V1. Importantly, our methods for obtaining responses and defining maps are nearly identical to these studies.

In Fig R4, we show the azimuth and altitude contours along with the derived borders for all the mice used in the paper (as also suggested by Reviewer 1, point 6 and Reviewer 2, point 6). Fig R4 is added to the paper as Supporting Information Fig S1.

Reviewer point 3.3 — *I am wary of the conclusions drawn from the unsupervised classification results. Specifically, it seems that the conclusions drawn result directly from the rules added to the unsupervised clustering. For instance, since clusters can only be merged if they are touching, it seems impossible for the lateral and medial areas to end up in the same clusters, especially given the 40% constraint, so it isn't clear to me how meaningful that result is. It is possible that the paper could stand without the unsupervised classification results. (See also Reviewer 1, point 12 and Reviewer 2, point 3)*

Response 3.3: As suggested by the reviewer, we carried our further analysis of unsupervised clustering. Our experiments and results are summarized in Response 1.12, and described in Appendix 1. We found little improvement, and have removed the analysis from the paper (as suggested above and by Reviewer 1, point 12).

Reviewer point 3.4 — *Throughout the paper, chance is said to be 1/6 given the six visual areas. It is not clear to me that this is the right level of chance to be used. Particularly for the widefield data, when more pixels are in V1 than any of the other visual areas, it seems that the prior should be shifted towards V1. Is there a way to define chance that takes the relative proportion of pixels (and neurons for the 2P data) for each of the areas?*

Response 3.4: As the reviewer suggested, we have included the proportion of pixels used for training as a bias to the random classifier. We show that the results are still better than the biased classifier. Only the supervised classifier (Section 2.1) uses training data in proportion to the available data from each area. In the semi-supervised classifier we start with equal amount of data for all the areas. The exact text added to **Results** (in Section 2.1) can be found below:

> To demonstrate the significance of results obtained in Tables 3 and 4 (in the main paper), we compared the results with two random classifiers. First, a random, unbiased six-faced die was considered. This random classifier will give a chance level accuracy of 16.67%, irrespective of the dataset. Secondly, we considered a six-faced die biased by the proportion of different area sizes (or the number of pixels/neurons used during training). For the wide-field dataset, this random classifier will give an average chance level accuracy of 37.6% (averaged across all five mice) and a maximum of 51.1% (for M1). Similarly, for dataset 2, this classifier will give an average chance accuracy of 26.7% and a maximum of 33.9% (for Nr5a1 Session C2). For both the datasets, the results obtained in Tables 3 and 4 were much higher than the random classifiers. These results suggest that the responses are discriminative between different areas.

Reviewer point 3.5 — *Related to this, I would like to see what the confusion matrix looks like for these classifications. Do mis-classified pixels(/neurons) tend to be classified as the closest area? To the area the best matches retinotopy for that location? Or do they default to V1?. (See also Reviewer 2, point 5)*

Response 3.5: In Fig R5, we show examples of confusion matrices, obtained using mouse M1 from wide-field dataset and EXM1-IRES Cre-line from two-photon dataset, respectively. In Supporting Information Fig S4, we show the same for the entire dataset. For the wide-field dataset, responses from other areas were mostly predicted as V1 (Fig R5), which is not unexpected since V1 projects to each of the other areas. The confusion observed in the two-photon dataset were variable. Importantly, however, for both datasets, the majority of neurons were predicted correctly. This has been added to the **Results** in Section 2.1.

Reviewer point 3.6 — *I would like to see a comparison of the semi-supervised area boundaries with retinotopy (eg. Fig 9 of Zhuang et al). It is not clear to me that the boundaries that this method is identifying do not reflect retinotopy. Eg. It appears that the semi-supervised boundaries separate altitude reasonably well (eg. the boundary between LM and RL that extends into V1 seems to match roughly with the horizontal meridian). The authors make the point that they are not using a retinotopic stimulus, but a natural movie stimulus has distinct information in different retinotopic locations – and thus could drive retinotopically distinct responses. If that is not true for the movies used in this study, I'd like to see an analysis to demonstrate the spatial/temporal content of the movies across retinotopy.*

Response 3.6: We show the results of semi-supervised clustering on natural movies, and three other grating stimuli responses (Section 2.2). In addition, we also show that resting state responses can be used to cluster different areas (Section 2.2). Hence, retinotopically distinct responses are not necessarily required to cluster the areas.

In Figs R6 and R7, we provide the spatial and temporal frequencies computed from different regions of the natural movie stimuli. The distributions of spatial and temporal frequency components are almost identical for different regions of the movies. Thus, it is very unlikely that different retinotopic locations were differently stimulated.

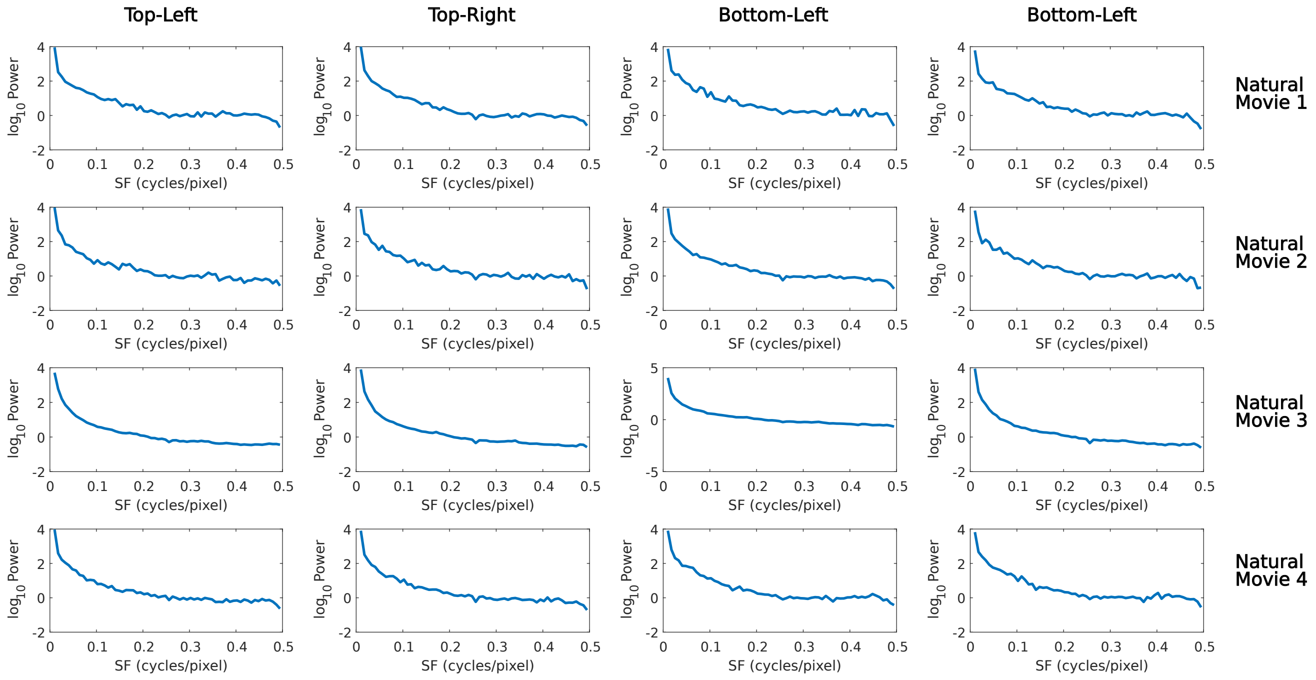

Fig R6. Distribution of spatial frequency computed from different regions of the natural movie stimuli used

Reviewer point 3.7 — *The biggest question that emerges for me from this work is what is the distinguishing features of the activity from the different areas. The authors conclude that these results suggest that each visual area has distinct response signatures, and some insight into the nature of those signatures would be very valuable. Particularly given that these classifiers can separate these visual areas using as little as a 4.5 second movie or even just 10 minutes of resting state activity. What are the features of the activity that the classifiers are using to separate these areas? An analysis of the classifier weights or features could be really illuminating in this regard. Or perhaps even example traces from the different areas. (See also Reviewer 1, point 1)*

Response 3.7: To follow up the reviewer's comment, we carried our further analysis by computing inter-areal and intra-areal correlations in wide-field and two-photon data. We argue that the supervised classifier is able to

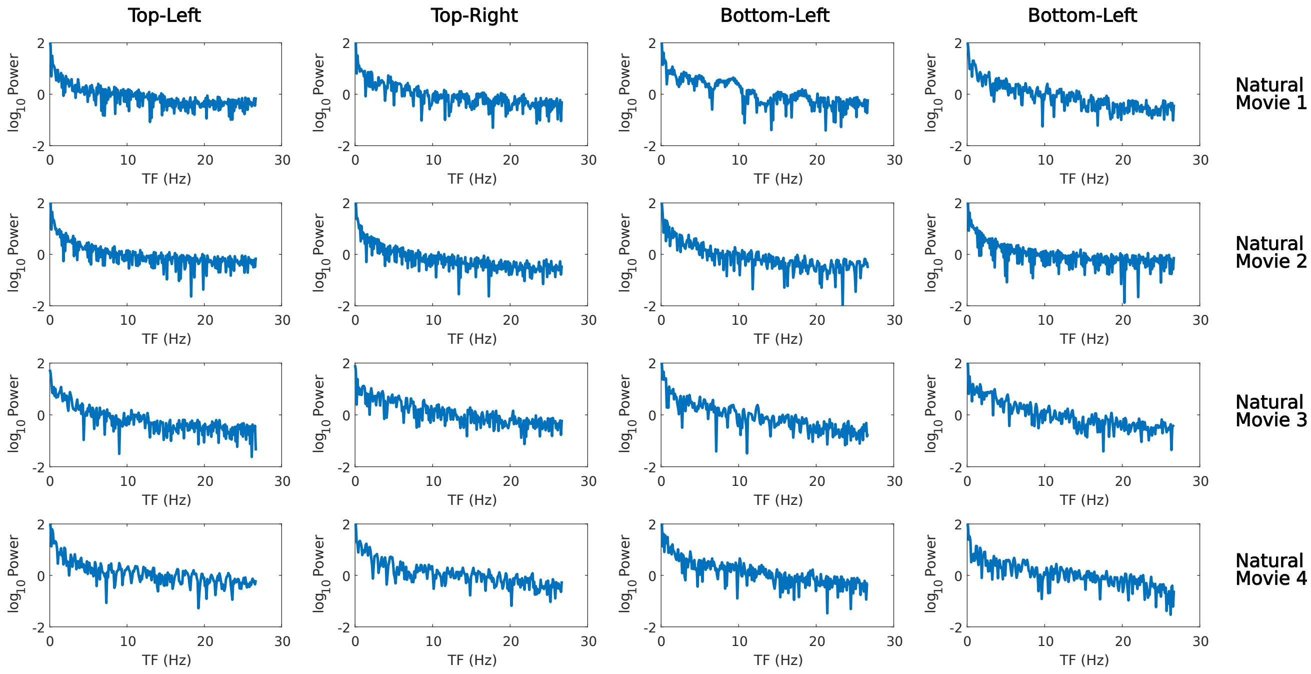

Fig R7. Distribution of temporal frequency computed from different regions of the natural movie stimuli used

cluster neurons from different areas using examples from the training data owing to stronger intra-area correlations. Response 1.1 and Figs R1 and R2 describe our analysis in detail. This analysis has been added to the **Discussion** (Section 3, Figs 8 and 9) in the paper.

Reviewer point 3.8 — *The analysis in Figure 6 comparing boundaries obtained with different durations of stimulus is very interesting and important. My concern is that the movie responses are averaged across trials while the resting state is not averaged. The nature of an averaged signal and an unaveraged signal is very different, so 20 seconds of average movie activity and 20 seconds of unaveraged resting state are not an equivalent comparison. Why not do the classification of movie responses without averaging the trials, thus allowing a direct duration comparison between the two?*

Response 3.8: We have changed Fig 7 (Figure 6 in the previous manuscript) and Section 2.3 completely in response to this comment. A summary of the changes is given below:

- We now extend the result in Fig 7 to supervised results as well. We also include results from the two-photon dataset of the Allen Institute.
- As the reviewer has suggested, we have also included single trial natural movie responses.

In the wide-field dataset, we observe that the single trial responses gave better accuracy than the resting state responses. However, with the two-photon dataset, the performance of background and single-trial movie responses was similar. For both the datasets, the trial-averaged responses gave better results than resting state responses.

Reviewer point 3.9— *Table 1 summarizing the stimuli used for widefield imaging needs more information. Namely the spatial and temporal frequencies and contrast used for stimulus 1. The directions, temporal frequency, and contrast for stimulus 2. The directions, spatial frequency, and contrast for stimulus 3*

Response 3.9: The missing information is shown in Table R1 (given as Table 1 in main paper). The table has been updated.

Reviewer point 3.10 — *Was the stimulus for the widefield imaging warped to account for viewing distance? Where was the monitor positioned relative the mouse's center of gaze? As different visual areas cover different regions of retinotopy, if the stimulus wasn't warped properly, the stimulus could have different content in different regions, and hence for different HVAs.*

Response 3.10: Visual stimuli were presented to head-fixed mice using a large display screen placed perpendicular to the right retina at an angle of $30°$ relative to the body axis of the animal, at an eye-to-screen distance of 12 cm. As mentioned in Section 1.1.1, display placement was ensured to cover as much of the contralateral visual field as possible.

We additionally did spherical warping of the visual stimuli during retinotopic mapping, using pschopy.visual.windowwarp function from Allen Institute. The rendering of stimuli was then manually checked to make sure the warping was effective.

Reviewer point 3.11 — *What was the mean luminance of the stimulus? Was the monitor gamma corrected?*

Response 3.11: The monitor was gamma corrected. The mean luminance was kept at $55\ cd/m2$. This information has been added to **Methods** (Section 1.1.1) in the text.

Reviewer point 3.12 — *What Cre line was used to drive the GCaMP6 expression in the widefield data*

Response 3.12: The Cre-line used in wide-field dataset was Emx1-IRES. This has been added to **Methods** (Section 1.1.1)

Reviewer point 3.13 — *How did the authors choose to analyze Emx1 and Nr5a1 from the Allen Brain Observatory dataset? (the authors mention these Cre lines were imaged across all six areas, but that is true for Cux2, Rorb, and Rbp4 as well – why were these not analyzed?) Was Emx1 used from all layers or only from specific layers?*

Response 3.13: The Emx1 Cre line was chosen because it is the same mouse line used for collecting wide-field data; here GCaMP is expressed only in excitatory cells. Nr5a1 was selected as an additional Cre line to complement our analysis. We now show the results for all other Cre lines, namely, Rorb, Cux2, Rbp4, and Slc17a7, which have recordings from all six areas in Table R4. Table R4 is added as Supporting Information Table S3.

Reviewer point 3.14 — *I'd like a bit more information about how the classifier was applied to the 2P data. Were all neural traces (for the chosen Cre lines) used, or subselected? If subselected, how was this done? What was the test/train split? Were equal numbers of neurons used for each area or was this different? How many neurons were used?*

Response 3.14: In the original manuscript, for each stimulus, the session was randomly picked from experiment containers in Allen Institute API. In the revised manuscript, for each stimulus, a particular session is fixed explicitly as detailed below.

The Allen Institute dataset has neuronal responses collected using four different session types. In each session, different stimuli were used. In the updated manuscript, we have used the data from "Session A" for natural movies 1, 3, and resting state responses. Also, we use data from "Session C2" for natural movie 2 responses. All the traces of particular stimuli were chosen from the given Cre line and session for the analysis. 50% of cells from each area were randomly selected from each area for training. Therefore, every area had a different number of cells for training. On fixing the number of training neurons from each area to the lowest, we observed a drop in performance. Despite the different number of training examples, confusion matrices in Fig R5 (given as Fig 4 in the main paper) show that the models produce a comparable precision for all the areas. These details have been added to **Methods** (Section 1.1.2) in the text. Table R2 (Table 2 in the main article) provides the number of neurons available for each area from the Allen Institute dataset. Classification accuracies in Table 4 (Table 3 in original manuscript) have been updated consequently.

Table R2: **Number of neurons available for analysis for each Cre-line and session from the Allen Institute dataset.**

Cre-line	AL	LM	RL	AM	PM	V1
Emx1-IRES (Session A)	1235	1446	1963	241	536	2199
Emx1-IRES (Session C2)	1148	1238	2085	226	552	964
Nr5a1 (Session A)	178	256	1074	110	203	441
Nr5a1 (Session C2)	106	267	1023	115	234	149

Reviewer point 3.15 — *Figure 3B shows generalization across mice. It's not clear to me whether this is to show similar results for different mice, or whether it is to show that training on one mouse can predict testing on a different mouse. I believe it's the former, but it would be very interesting if it were the latter. Please clarify. (See also Reviewer 2, point 7)*

Response 3.15: In Fig 3B, by generalization across mice we mean that the classifiers work similarly for different mice when trained and tested individually. We tried pooling data from mice M1 and M2. When training data is

sampled from this pooled dataset, the classifier worked with accuracy of about 90% for test data, from both M1 and M2. However, when trained on M1 and tested on M2, the result did not scale. This shows that each mouse's data is quite variable, and the proposed classifier only works for the mice that were seen during training. Indeed, it would be very exciting to train a model that could be generalized across mice, as this would tease apart the covarying factors from invariant factors in the area-area relationships. However, it is not clear whether such invariant relationship exist at this level. Further studies are needed to address this important question.

Reviewer point 3.16— *Figure 4 color labels appear to be mis-assigned*

Response 3.16: The labels has been fixed in Fig 4D

Reviewer point 3.17 — *Why is the accuracy for widefield pixels so much higher than for the 2P neurons? Given the shorter movie clip, and the single pixel data, I'd expect the widefield data to perform worse than 2P, not better. But perhaps the fact that the widefield signal for a pixel could combine activity from multiple neurons/processes could play a role in this? In a similar vein, why do Emx1 and Nr5a1 perform differently? Are there different numbers of neurons available? Could it be layer specific? I don't think these can necessarily be conclusively answered, but if possible some discussion of these questions would help.*

Response 3.17: We believe the wide-field pixels work better owing to the spatial correlation among the pixels in the dataset. To counter this effect, in Section 2.1, we sample training data from only the center of each area. In Section 2.2, we propose semi-supervised clustering, which uses only the minimum amount of data from each area. As we make the problem more constrained in the wide-field dataset, we observe the accuracy to drop. However, even in this constrained setting, the accuracy obtained is always significantly better than the chance level.

In the revised manuscript, we have fixed the session for each stimulus to enable better comparison (See Reviewer 3, point 14). In addition to two Cre-lines in the main paper, we now show the results for all other Cre-lines, namely, Rorb, Cux2, Rbp4, and Slc17a7, which have recordings from all six areas in Table R4. The number of neurons available for two Cre-lines used in the paper is shown in Table R2 (Table 2 in the main paper). The same information for the additional four Cre-lines are shown in Table R3. We do observe that the result varies with each Cre-line, irrespective of the total number of neurons. However, for all the Cre-lines, the results are consistently higher than the accuracy of a random classifier (see Reviewer 3, point 4). Tables R3 and R4 are added as Supporting Information Tables S2 and S3.

Table R3: **Number of neurons available for other Cre-lines in the Allen Institute dataset.** The classification accuracies are shown in Table R4

Cre-line	AL	LM	RL	AM	PM	V1
Slc17a7-IRES2 (Session A)	223	2184	97	138	1245	4499
Slc17a7-IRES2 (Session C2)	267	2140	70	149	1220	4232
Rorb-IRES2 (Session A)	794	767	975	516	511	1617
Rorb-IRES2 (Session C2)	292	421	1082	404	397	515
Cux2-CreERT2 (Session A)	2219	1820	1271	1060	1624	3558
Cux2-CreERT2 (Session C2)	497	358	1275	871	289	774
Rorb-IRES2 (Session A)	267	333	72	244	375	320
Rorb-IRES2 (Session C2)	109	194	68	237	141	242

Reviewer point 3.18— *The Allen Brain Observatory is from the Allen Institute for Brain Science (not Allen Brain Institute – line 42). The citation should also match the citation policy for the dataset (https://alleninstitute.org/legal/cite policy/)*

Response 3.18: Citation for the dataset from the Allen Institute has been corrected in the updated paper. The dataset is referred to as from the Allen Brain Observatory, Allen Institute for Brain Science, or the Allen Institute dataset in short.

Table R4: **Classification accuracy for other Cre-lines in the Allen Institute dataset.** The results are averaged across random initializations. The entries denote "*% accuracy (± standard deviation)*"

Cre-line (Session)	Stimuli	Accuracy of Supervised Classifier			
		GMM	SVM	ANN	Bayes
Slc17a7-IRES2 (Session A)	Natural Movie 1	58.0 (±1.28)	65.4 (±0.49)	65.0 (±0.577)	57.10 (±0.98)
	Natural Movie 3	68.6 (±1.44)	71.0 (±0.26)	71.4 (±0.13)	66.9 (±0.82)
	Resting State	55.9 (±1.13)	61.2 (±0.85)	59.9 (±1.08)	44.6 (±1.68)
Slc17a7-IRES2 (Session C2)	Natural Movie 2	59.0 (±1.01)	63.0 (±0.33)	62.8 (±0.50)	55.7 (±0.59)
Rorb-IRES2 (Session A)	Natural Movie 1	42.2 (±1.44)	45.2 (±0.68)	44.9 (±0.78)	42.0 (±0.47)
	Natural Movie 3	48.5 (±1.77)	53.0 (±0.30)	53.4 (±0.57)	48.7 (±0.84)
	Resting State	65.3 (±1.62)	67.8 (±1.05)	68.4 (±1.64)	63.5 (±1.80)
Rorb-IRES2 (Session C2)	Natural Movie 2	46.8 (±0.59)	48.3 (±0.78)	47.6 (±0.57)	44.6 (±1.31)
Cux2-CreERT2 (Session A)	Natural Movie 1	37.5 (±1.16)	43.0 (±0.68)	43.5 (±0.70)	39.3 (±0.72)
	Natural Movie 3	39.0 (±0.82)	47.7 (±1.27)	50.1 (±0.93)	38.7 (±1.09)
	Resting State	45.8 (±1.15)	48.6 (±0.98)	47.3 (±1.005)	39.2 (±0.69)
Cux2-CreERT2 (Session C2)	Natural Movie 2	45.6 (±1.47)	46.9 (±1.66)	46.4 (±1.70)	44.9 (±1.30)
Rbp4 (Session A)	Natural Movie 1	34.6 (±2.20)	34.6 (±1.88)	34.5 (±2.17)	33.1 (±2.00)
	Natural Movie 3	38.1 (±2.50)	39.7 (±2.23)	38.2 (±2.31)	35.9 (±2.27)
	Resting State	35.6 (±1.79)	41.0 (±2.73)	39.9 (±2.92)	33.7 (±2.75)
Rbp4 (Session C2)	Natural Movie 2	34.0 (±1.64)	34.2 (±1.09)	32.5 (±1.51)	31.4 (±0.81)

Appendix 1 Revised experiments on unsupervised clustering of mouse visual cortex

The unsupervised approach is a hierarchical clustering technique very similar to the semi-supervised approach. The clustering starts with an initial division of the visual cortex into chunks of uniform size. Similar to semi-supervised clustering, the size of the initial chunks is a hyperparameter to be set empirically. In every iteration, only the neighboring clusters with highest score are merged. BIC and the determinant of covariance of the merged cluster are used as two different metrics for the clustering algorithm.

$$\mathrm{S}_{a,b} = log\ p(D|\boldsymbol{\lambda}) - (\ log\ p(D_a|\boldsymbol{\lambda}_a)\ +\ log\ p(D_b|\boldsymbol{\lambda}_b)\ ) \tag{R1}$$

For computing BIC, unimodal Gaussian distribution are fitted to every initial cluster. The BIC score provided in Equation R1 is used to evaluate neighboring clusters for merging. After merging, the new cluster is modeled by a GMM with the number of components as the sum of the number of Gaussians in separate clusters. This technique is analogous to unsupervised clustering of speakers in Ajmera and Wooters [2003]. Since the total number of parameters is constant throughout the clustering process, in Ajmera and Wooters [2003], the clustering is stopped when the BIC score is ≤ 0 for all the possible merges. When the same stopping criteria are applied to the visual cortex, the clustering ends after a few iterations. Hence, the clusters with highest BIC score are merged until there are only six clusters left, which is the count of visual areas studied in the supervised approaches.

The determinant of covariance matrix is used as another score to merge clusters. This score is measure of a generalized variance in the cluster. The two neighboring clusters having the lowest value of the determinant are merged in every iteration.

In addition to the above metrics, two physiological rules, derived empirically from the observed maps were added to the clustering approach.

Rule 1: A single cluster cannot be larger than 40% of the total number of clusters.

Rule 2: One visual area cannot morphologically enclose another.

Rule 1 prevents the formation of a single dominating cluster. The primary visual cortex (V1) being the largest visual area, occupies about 40% of the total area of the visual cortex. When the merged cluster size exceeds this threshold, the cluster pairs with next highest BIC score are chosen for merging. Hence this rule limits the maximum size of a cluster to the size of V1. Rule 2 is a de facto condition that is checked while finding the visual areas from the retinotopic maps in Garrett et al. [2014]. In Fig R8, an illustration of the unsupervised clustering is shown. During the process of merging, if a cluster is found to enclose another, it is morphologically closed. The sequence of steps for clustering is presented below:

Step 1: The dimensions of the wide-field responses are reduced using PCA (as described in Methods (Section 1.2.1)).

Step 2: The visual cortex is initially divided into square grids of equal size. Every cluster is modeled by a unimodal Gaussian distribution. The parameters are estimated using maximum likelihood estimation.

Step 3: BIC (Equation R1) or determinant of covariance matrix is computed as score between every pair of adjacent clusters. Two clusters that share a boundary are defined as neighboring clusters.

Step 4: The pair with best score is merged into a single cluster. The merged cluster are modeled by a GMM with the number of components as the sum of the number of Gaussians in separate clusters.

- Step 4.1: The merged cluster is checked for rule 1. If the merged cluster size is higher than 40% of the total visual cortex size then the cluster pair with next best score is merged.
- Step 4.2: The merged cluster is checked for rule 2. If the merged cluster completely encloses another cluster then the former is morphologically closed.

Step 5: Steps 3 to 7 are repeated until the total number of unique clusters are six. Finally, to smoothen the boundaries, a supervised classifier is trained by sampling from the final clusters.

The results of clustering the visual cortex using the unsupervised pipeline using BIC is given in Fig R9. The results using determinant of the covariance matrix as the merging criteria is given in Fig R10. Unlike the supervised approaches, there is no one to one mapping between the clusters obtained and the visual areas identified by the retinotopic procedure. Hence the final clusters are evaluated using V-measure (Rosenberg and Hirschberg [2007]) instead of accuracies. V-measure is computed as the harmonic mean of completeness and homogeneity of the obtained clusters.

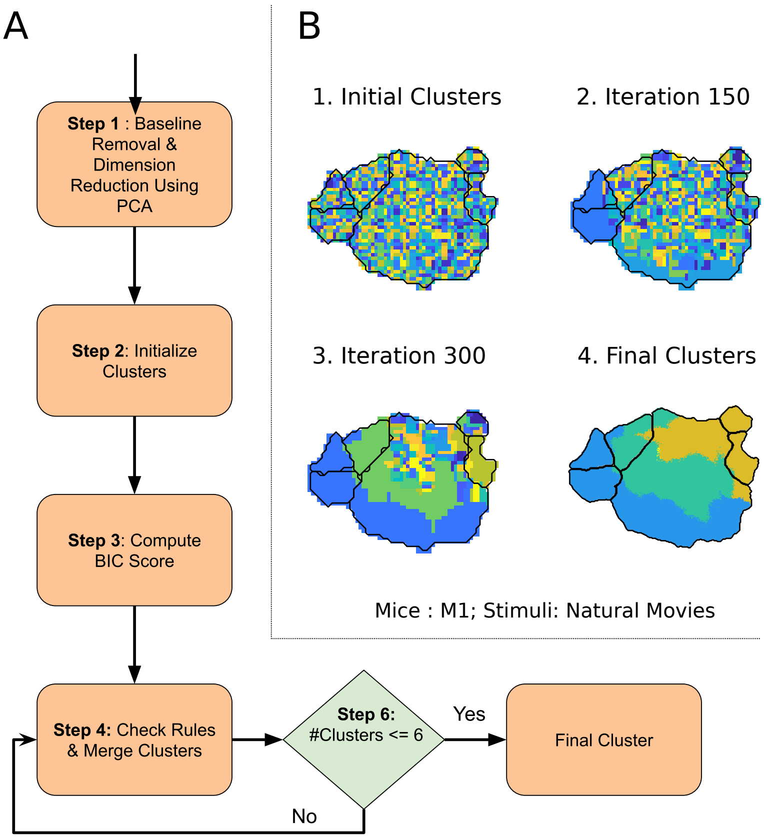

Fig R8. Illustration of unsupervised clustering. A) Block diagram of the unsupervised clustering pipeline. B) The clustering process is shown through various iterations along with the final cluster for Natural Movie stimuli.

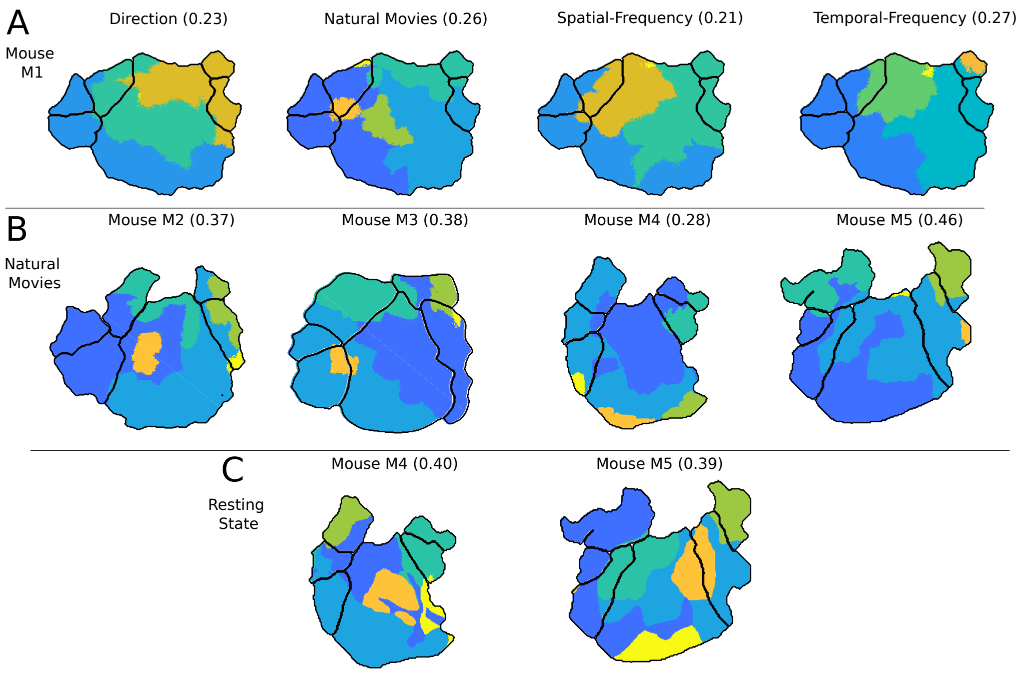

Fig R9. Unsupervised clustering of mouse visual cortex using BIC. A) Clusters obtained for different visual stimuli in mouse M1. The visual stimuli are as mentioned above each plot. **B**) Clusters obtained for different mice using natural movies as stimuli. **C**) Clusters obtained from resting state responses in two different mice. The values in bracket denote the V-measure (Rosenberg and Hirschberg [2007]) of the clustering result.

In Figs R9 and R10 the results are shown for different mice as well as different visual stimuli, and resting state. Although the final clusters vary across mice and stimuli, some properties are consistent across various mice. From

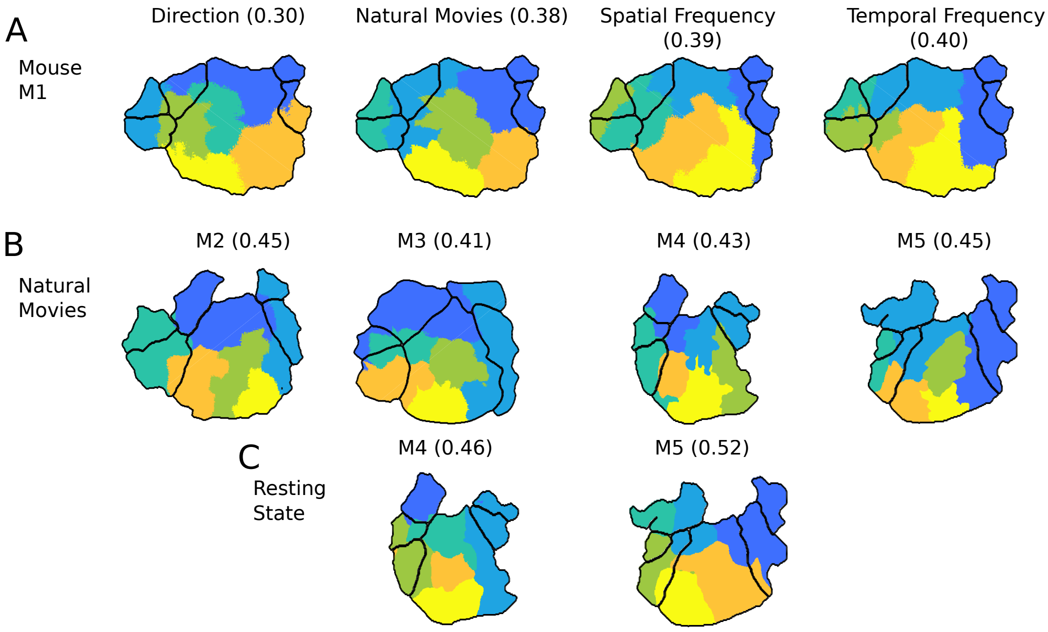

Fig R10. Unsupervised clustering of mouse visual cortex using determinant of the covariance matrix. A) Clusters obtained for different visual stimuli in mouse M1. The visual stimuli are as mentioned above each plot. **B)** Clusters obtained for different mice using natural movies as stimuli. **C)** Clusters obtained from resting state responses in two different mice. The values in bracket denote the V-measure (Rosenberg and Hirschberg [2007]) of the clustering result.

the results, the following properties are observed consistently (i) The lateral areas (LM, AL, and RL) are always modeled by different clusters when compared to the medial areas (AM and PM). (ii) Areas LM and AL appear to be consistently clustered together. This observation indicates that there is a difference in the signatures of lateral and medial areas. In most of the cases, V1 seems to be split by the lateral and medial clusters, suggesting that the signatures of V1 are slightly different from that of the lateral and medial areas. These observations are consistent with resting state responses as well. A better merging criterion is required to cluster these areas effectively. The obtained results in Figs R9 and R10 are still very different from the retinotopically defined areas and hard to interpret precisely. Hence these results have been removed from the main article.

References

J. Ajmera and C. Wooters. A robust speaker clustering algorithm. In *IEEE Workshop on Automatic Speech Recognition and Understanding*, pages 411–416. IEEE, 2003.

M. L. Andermann, A. M. Kerlin, D. K. Roumis, L. L. Glickfeld, and R. C. Reid. Functional specialization of mouse higher visual cortical areas. *Neuron*, 72(6):1025–1039, 2011.

S. Elhabian and A. A. Farag. A Tutorial on Data Reduction: Linear Discriminant Analysis (LDA). http://www.sci.utah.edu/~shireen/pdfs/tutorials/Elhabian_LDA09.pdf, 04 2009. (Accessed on 6/9/2020).

M. E. Garrett, I. Nauhaus, J. H. Marshel, and E. M. Callaway. Topography and areal organization of mouse visual cortex. *Journal of Neuroscience*, 34(37):12587–12600, 2014.

L. v. d. Maaten and G. Hinton. Visualizing data using t-sne. *Journal of machine learning research*, 9(Nov): 2579–2605, 2008.

R. V. Rikhye and M. Sur. Spatial correlations in natural scenes modulate response reliability in mouse visual cortex. *Journal of Neuroscience*, 35(43):14661–14680, 2015.

A. Rosenberg and J. Hirschberg. V-measure: A conditional entropy-based external cluster evaluation measure. In *Proceedings of the 2007 joint conference on empirical methods in natural language processing and computational natural language learning (EMNLP-CoNLL)*, pages 410–420, 2007.

J. Waters, E. Lee, N. Gaudreault, F. Griffin, J. Lecoq, C. Slaughterbeck, D. Sullivan, C. Farrell, J. Perkins, D. Reid, D. Feng, N. Graddis, M. Garrett, Y. Li, F. Long, C. Mochizuki, K. Roll, J. Zhuang, and C. Thompson. Biological variation in the sizes, shapes and locations of visual cortical areas in the mouse. *PLOS ONE*, 14(5): 1–13, 05 2019. doi: 10.1371/journal.pone.0213924. URL https://doi.org/10.1371/journal.pone.0213924.

H. Yao, L. Shi, F. Han, H. Gao, and Y. Dan. Rapid learning in cortical coding of visual scenes. *Nature neuroscience*, 10(6):772–778, 2007.

J. Zhuang, L. Ng, D. Williams, M. Valley, Y. Li, M. Garrett, and J. Waters. An extended retinotopic map of mouse cortex. *Elife*, 6:e18372, 2017.
