# Supplementary material for: Functional parcellation of mouse visual cortex using statistical techniques reveals response-dependent clustering of cortical processing areas
Source: PLoS Comput Biol. 2021 Feb 4;17(2):e1008548. doi: 10.1371/journal.pcbi.1008548 (PMC7888605; doi:10.1371/journal.pcbi.1008548)
Supplement: Attachment — Submitted filename: response_to_reviewers.pdf [file pcbi.1008548.s012.pdf]

Response to reviewers

“Functional Parcellation of Mouse Visual Cortex Using Statistical Techniques Reveals Response Dependent Clustering of Cortical Processing Areas” (PCOMPBIOL-D-20-00156)

We thank all three reviewers and the editors for valuable feedback on our paper. A point-by-point response to the comments/concerns expressed by Reviewer 1 and 3 are given below.

Reviewer 1

Reviewer point 1.1

I remain convinced that the visual area borders are erroneous. The paper reports differences across identified visual areas so alignment to previously established borders is critical. I and the other reviewers all raised concerns about the maps and this problem needs to be resolved if this paper is to be meaningful. The problem is less obvious now that there are no sign maps in the revised paper. The lack of sign maps, on which the border assignments are based, is a problem. I suggest the authors add field sign maps, with borders marked, to Fig R4 and add figure R4 to the paper. The more fundamental problem remains that border locations are not where the field sign crosses zero. Borders at zero-crossing is basis field sign mapping. The authors’ claim that they have duplicated the field sign mapping approach of previous papers is inaccurate – this much is clear from their figures. To illustrate the problem, below I’ve reproduced a panel from figure 1 of the original submission. Field sign = 0 is in the light blue/green color zone. The borders are generally not in the light blue-green zone and so at not at field sign = 0. Why is the border not at field sign = 0? The error needs to be found and resolved. The resulting shift in border positions may affect the results and conclusions.

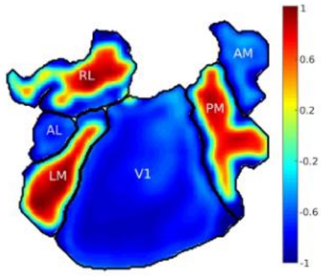

Response 1.1

We thank the reviewer for this comment. We have now carefully reviewed all the boundaries and have verified them to be correctly aligned to the sign map. As the reviewer has requested, we now show the sign map along with the horizontal and vertical retinotopy contours for all experimental animals in Fig R1. Fig R1 is also updated as new Supporting Information figure S1 in the text.

As stated earlier, the final boundaries were formed using post-processing (filtering, thresholding/binarization and moropological processing) over these continues sign maps (similar to (Garrett, et al., 2014)). The code used to generate the sign map and the borders is publicly available (Garrett, et al., 2014) and can be downloaded from https://labs.la.utexas.edu/nlab/imaging-visual-cortex-matlab-code/.

In Fig R1, we found that for mouse M5, the sign map computed now was different from the one plotted in Fig 1D of the original submission. We went back and did a thorough analysis of how Fig 1D was generated in the original submission. We found an error in the post-processing of sign maps just before they were included in the original submission. We apologize for this oversight, and explain in detail below what went wrong:

1. Author-2 collected the dataset 1, computed the sign maps, and subsequently, the boundaries. The sign map was then post-processed and included in Fig 1D by Author-1.
2. The thresholded sign map computed by Author-2 is shown as Fig R2-A. Notice that areas with a positive sign have values in the range [1.5, 2], and areas with a negative sign have values [0.25, 0.75]. The boundaries or pixels where sign change occurs corresponds to the value 0. Author-2 made this kind of representation for better visualization

3. Author-1 assumed the sign map continued having values in the range -1 to +1 and applied a Gaussian smoothing to filter out the noise. This figure is shown as Fig R2-B. Later this smoothed map was re-scaled between -1 and +1 and plot as Fig 1-D in the original submission. This figure is shown as Fig R2-C.
4. The negative sign areas got more smoothed since the value was closer to zero than the positive sign areas (see point 2 and scale in Fig R2-A). Owing to the misunderstanding in the sign map representation, the correctly computed border became misaligned. In other words, the pixels corresponding to the sign change got merged with the areas having a negative sign.
5. That said, this erroneous representation of the sign map that was given in Fig 1-D was not used further to arrive at the area boundaries. The correct sign map, along with the horizontal and vertical retinotopy contours, are given in Fig R1.

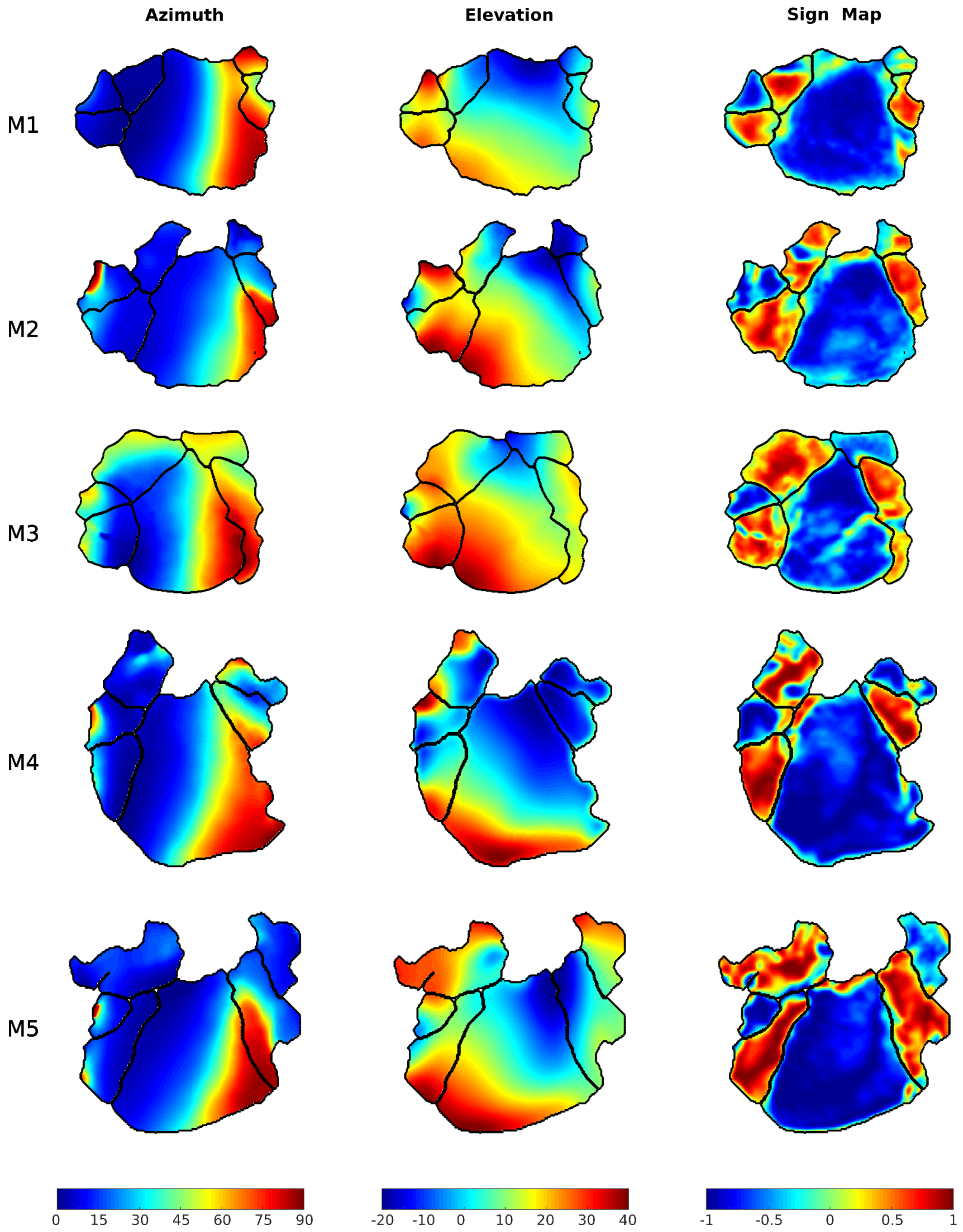

Fig R1 Horizontal and vertical retinotopy along with sign map within six visual areas of all the mice used in the paper. Cortical areas of the left hemisphere are shown. Azimuth 0º and 90º correspond to the midline and the far periphery of the contralateral visual field, respectively. Negative values of elevation represent lower visual field, and positive values represent upper visual field.

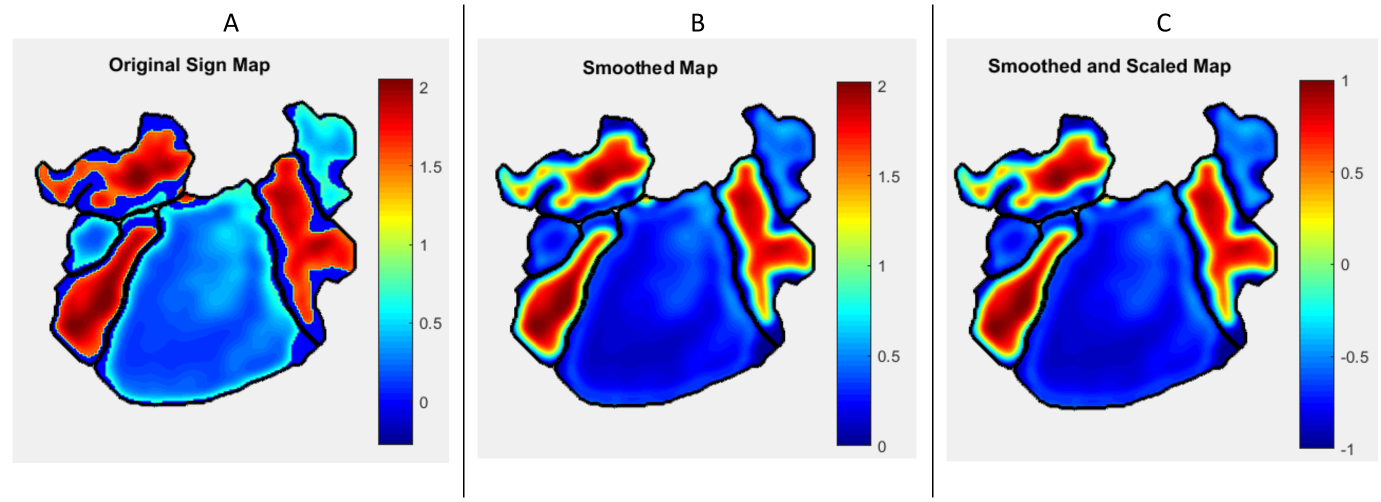

Fig R2 Illustration of how the sign map was computed for Fig 1D of the original submission

Reviewer point 1.2

Where are the cluster-based borders and how do they relate to borders identified in previous papers? Figure 9 indicates that some pixels from V1 cluster with pixels from LM, AL, PM and RL. I've indicated these pixels in the figure below, copied from the updated paper. Where are these pixels? Are they along the interface between V1 and adjacent areas? If they are, clustering predicts slightly different border locations that the field sign mapping procedure. Of course, field sign mapping predicts slightly different borders to architectonic methods (Zhuang et al., 2017). Do the cluster borders more closely match the field sign borders or architectonic borders, or does clustering identify a third set of borders? Answering these questions would link the paper directly to the existing literature.

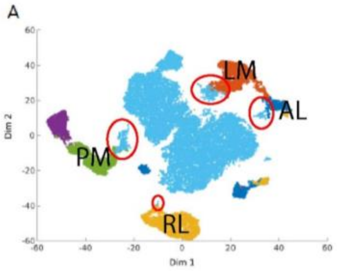

Response 1.2

Firstly, we clarify that in Fig 9, using tSNE, we present a visualizable 2D representation of the high dimensional LDA features. The high dimensional LDA features were used by the classifiers in Section 2.1 to predict the boundaries. In the text, we have already compared the cluster borders obtained with the retinotopic border (Fig 3). In Fig 4, we also show the confusion matrix to explain the misprediction in various areas.

In Section 2.2, we use a semi-supervised clustering algorithm to arrive at the boundaries. In Fig 6, we compare the predicted boundaries with the retinotopic boundaries. We find the boundaries predicted by the supervised approach are aligned more with retinotopic boundaries than the semi-supervised boundaries. Hence, we have already compared all the predicted boundaries with retinotopic boundaries in the text.

As mentioned earlier, Fig 9 just shows a possible 2D representation (obtained using tSNE) of the LDA features for test data. The only conclusion that we can make from Fig 9 is that most pixels from each area are clustered together. The tSNE is an algorithm proposed for rough visualization of high dimensional data (Maaten & Hinton, 2008). tSNE primarily attempts to preserve the structure of the data in the higher dimensional space. The 2D representation cannot be considered precise as it can vary depending on a random initialization (Kobak & Berens, 2019). Our tSNE plot is aimed at visualizing the preserved local structures (mainly in terms of clusters) in the data. These local structures correspond more to functional similarities rather than anatomic proximity. In other words, pairs of pixels that lie farther apart anatomically may be embedded closer in tSNE space if they are functionally (in terms of average responses to visual stimuli) similar than those that are anatomically closer but have distinct responses (Yildirim, et al., 2020). Thus, we do not expect that tSNE cluster borders match faithfully with either retinotopic borders or architectonic borders.

Reviewer 3

Reviewer point 3.1

The addition of the confusion matrix in Figure 4 is valuable and informative – thank you for adding that. It might be worth pointing out that for the widefield data, ignoring V1, there is a higher (though not high) confusion of AL and LM for each other, and of AM and PM for each other. To me, this is interesting, and is consistent with other studies that show these two pairs of areas have more similar responses with each other that with other areas. This might be worth mentioning as I believe that it supports the idea that the clustering here represents functional differences. The fact that this is not clear in the 2P data, however, might make this harder to support. Could you comment on why the confusion is greater for the 2P than the widefield? Is it that there is diversity of functional responses of neurons, so when looking at individual neurons the confusion will be higher than pixels that are merging signals from multiple cells?

Another question I have from the confusion matrix is that in the 2P data particularly, areas AM and PM appear to have lower accuracy than the other areas. Perhaps this is more true for the Emx1 data than the Nr5a1 data (the lower and more variable # of neurons in the Nr5a1 data makes me a bit wary of over-interpreting those results). Could you speak to why this might be?

Response 3.1

In Section 2.1, we now mention that, following V1, the areas AL, LM, and AM, PM are observed to have the next highest confusion. This result is consistent with previous studies suggesting that these areas may constitute different processing streams (Marshel, Garrett, Nauhaus, & Callaway, 2011) (Wang, Sporns, & Burkhalter, 2012) (Juavinett & Callaway, 2015) (Smith, Townsend, Huh, Zhu, & Smith, 2017).

The reviewer correctly points out that for the two-photon dataset, the confusions are different and higher than the wide-field dataset. Also, in the two-photon dataset, the true-positive rates for each area were variable. There are two main reasons for this:

1. As the reviewer suggested, the physiology of the signal is different between two-photon and wide-field imaging. The pixels represent the cumulated response of neurons; the two-photon dataset captures the responses of individual neurons. Hence, there can be a higher variability in the neuronal responses, which leads to poor performance.
2. We have selected all the neurons available for the given Cre-line. However, this hardly represents the entire population response from the area, as only a few neurons are recorded from each area, and they are pooled from different mice.

These reasons have now been added to Discussion (Section 3) in the text.

Reviewer point 3.2

I continue to disagree with the authors' claim that the natural movie stimulus does not contain retinotopic information. This is not true. For any given frame of the movie, there is different content in different retinotopic regions. This might average out across all the frames so that the spatial/temporal frequency content is similar across space, but for each frame it is different. This is also true of the stimulus used for retinotopic mapping. While across the entire stimulus the content across space is the same, for each frame it is different – and THIS is what enables the authors to use the stimulus to map the retinotopy of the mouse visual cortex. But the same feature in the movies, different content in different regions of space for each individual frame, means that the movies do contain retinotopic information. The authors claim the opposite which is not true. This must be fixed.

Response 3.2

We believe the reviewer is referring to the following line from Section 2.1 in the text.

> | "The wide-field/two-photon calcium response for any given visual stimulus was first averaged across trials and converted to a lower dimension space using PCA followed by LDA (Section 1.2). **It is to be noted that the stimulus sets used here (Section 1.1) have no retinotopic information**."

By the term "retinotopic information", we mean to convey that the input data to our algorithm does not have explicit retinotopic information of which pixels respond to which azimuth and elevation in the visual field. To derive the visual area boundaries, this explicit retinotopic information is used. We have now corrected this statement in Section 2.1 as given below

> | "The wide-field/two-photon calcium response for any given visual stimulus was first averaged across trials and converted to a lower dimension space using PCA followed by LDA (Section 1.2). **It is to be noted that the stimulus information was only used to average across trials, and no other explicit information about the stimulus**

configuration or retinotopy was given as input (we cannot exclude the possibility that stimuli such as natural movies contain implicit retinotopic information)."

We agree with the reviewer that there will be differences in spatial/temporal content in each frame across the visual field for the natural movie stimulus; hence, the response contains implicit retinotopic stimulation. Importanly however, our methods don't use explicit retinotopic information.

Reviewer point 3.3

And I still would like to see the comparison of the semi-supervised clustering with retinotopy (eg. Fig 9 of Zhuang et al) as the area borders appear to match retinotopy better than the area boundaries (that are derived from retinotopy).

Response 3.3

In Fig 6 of the text, we compare the boundaries predicted by semi-supervised clustering with the retinotopically defined boundaries. We observe the final clustering boundaries are slightly shifted from the retinotopic boundaries, and we were able to obtain classification results of about 70% for all five mice.

In Fig R3, we now show the boundaries obtained by the semi-supervised clustering algorithm along with the horizontal and vertical retinotopy. We also show the boundaries derived from the retinotopic mapping. We observe that the borders derived from the retinotopic maps match the retinotopy better than the semi-supervised approach, at least qualitatively.

Reviewer point 3.4

I think Figure 7 improves the previous analysis of stimulus duration and enables a better comparison between the movie and resting state results. I think the authors could use this to emphasize two things in their text:
First, one of the stunning things from this analysis is that only a very short movie is needed to get fairly accurate separation of visual areas in the widefield data. 4.5 seconds contains probably only a few hundred stimulus frames, and they are likely fairly correlated frames at that. The fact that just a few visual stimulus features can drive this level of accuracy is, to me, really surprising, and I think this could be emphasized more in the text.
Second, the difference in the results here for supervised vs semi-supervised, and between widefield and 2p datsets, could be valuable for helping to understand the differences between these datasets. Eg. The fact that 2p accuracy is higher for averaged movie responses than single trial, while widefield shows little difference between them, could point to the impact of the correlated pixels on the results (eg. averaging across pixels vs averaging across trials). I think the fact that the supervised clustering (both widefield and 2p) has similar performance for resting state and single trial movie, while semi-supervised shows a difference between the two, might be similarly revealing for understanding the differences in those methods/results.

Response 3.4

For the first part, as the reviewer suggested, we have highlighted in the text (Section 2.3) that the supervised classifiers were able to cluster the areas just using 4.5 secs of response.

For the second part, see our response to Reviewer point 3.1, where we have detailed the difference between dataset 1 and 2. We now have included this difference as a part of the Discussion (Section 3) in the text.

Reviewer Point 3.5

I find the new analysis in section 3 (figures 8 &9) very confusing and I don't know that it helps to address the question that I had in my previous review.
First, the comparison of inter- and intra-areal correlations for the 2P dataset is fraught with problems. Namely, for the inter-area correlations, it must be pointed out (based on my understanding of the 2P dataset) that the areas are imaged in different sessions and different mice, while the intra-areal correlations include at least some data collected within the same session. This will make the intra-areal correlations higher simply because factors such as running or brain state will be in common. And while the correlations in the context of the movie at least have a common stimulus, the correlations of resting state … I just don't know how to think about that comparison of inter- and intra-areal correlations. I would recommend the authors not include the 2P dataset in this particular analysis because of these issues.

However, my larger concern is that this analysis does not address my original question of what features of the activity are that separate these areas. The authors claim in their reply that this analysis shows that the intra-correlations are the key features. First, this analysis doesn't convince me of this. I fully expect neighboring pixels/neurons to be more correlated, if only because they are retinotopically adjacent and thus likely receive common inputs, etc. Even in the absence of a

patterned stimulus, they should have more correlations. I'd be more convinced if the inter- vs intra- areal correlations took retinotopy into account – eg. compared the same regions of retinotopy across area.

However, even then, if the answer that the authors have for what features of activity separate the areas is just higher correlations within areas than across areas, it doesn't tells us what's different about, eg., AL and AM. The authors make the conclusion that "visual cortical areas have characteristic activity patterns" – and to me this statement is not supported by this comparison of correlations. It's not clear to me that this type of analysis is unable to address the question of what distinguishes the areas, in which case I think the authors should walk back such claims.

Response 3.5

The reviewer has raised three important concerns on the newly added correlation analysis, which can be summarized as follows:

1. Correlations in the two-photon dataset have been computed using multiple sessions and mice
2. Suggestion to use the same regions of retinotopy across areas to compute and compare the inter- and intra-area correlations
3. Higher intra- over inter-area correlation does not help to conclude that "visual cortical areas have characteristic activity patterns."

Below, we address each of these concerns in detail.

Correlations in the two-photon dataset have been computed using multiple sessions and mice: As the reviewer notes, the two-photon dataset was collected from different mice and sessions. The two-photon dataset analysis utlized multiple mice and sessions because we aimed to study all the six prominent areas in the mouse visual cortex. In Fig R4, we show the intra- and inter-area correlations for the two-photon dataset, computed from ten individual mice for natural movie stimuli, and in Fig R5 the correlations from the same mice for resting-state responses is shown. Fig R4 and R5 show that even at the level of individual mice, the intra-area correlations are higher than inter-area correlations. In Fig R4 and R5, we also mention the session number in which the data was collected. Different sessions had a unique set of neurons from the imaged area. In Fig R4-F to G, it can be seen that even across different sessions, the intra-area correlation is still higher than the inter-area correlation. In Fig R5-F to G, we do not find a high intra-area correlation across a different session. This result is expected since these are uncontrolled signals and have no relation across sessions.

Because two-photon data from different areas were collected from different mice at different times, it is likely that inter-areal correlations would be lower than intra-areal correlations. Comparing responses across different sessions shows reduced inter-areal correlations, but intra-areal correlations remain high for natural movie stimuli though not for resting state responses (Fig. R4 and R5). These findings are consistent with our conclusion (Discussion, section 3) that intra-areal correlations reflect unique patterns of circuits in each area, and visual stimuli are stronger drivers of internal circuits than resting state responses.

We thank the reviewer for highlighting this issue. To make this difference between the wide-field and two-photon dataset clear to readers, we now include Fig R4 and Fig R5 as Supplementary Information figures S5 and S6. We also discuss this difference between the two datasets in Section 3 (Discussion) of the text.

Suggestion to use the same regions of retinotopy across areas to compute and compare the inter- and intra-area correlations

In Fig R6-A and C, we show the intra and inter-area correlations computed using all pixels. In Fig R6-B and D, we show the corresponding correlations computed using patches of pixels with approximately the same retinotopy from each area and averaged across the entire visual space. Both Fig R6-A, C, and Fig R6-B, D, show that the intra-area pixels are more correlated than the inter-area pixels.

The proposed classification or clustering pipeline never used the retinotopic information directly. The retinotopic information was only used to derive the boundaries, which was used as ground truth for data-driven models. In Fig 3 of the text, we show that the proposed classifier works even with training pixels sampled from the center of each area, which does not retinotopically sample the entire visual field. Further, semi-supervised classifiers (Section 2.2) are shown to work using a patch of pixels from the center. Since we have never used the retinotopic information explicitly in our pipeline, we believe retinotopic information is employed indirectly at best in the classification. We have added Fig R6 as Supplementary Information figure S9 to the text.

Higher intra- over inter-area correlation does not help to conclude that "visual cortical areas have characteristic activity patterns"

For both the dataset, we have used the raw responses as input and applied PCA and LDA as dimension reduction techniques. For the two-photon dataset, the analysis was done at the neuron level, while that done for the wide-field dataset was done at the pixel level. The pixel-level activity corresponds to a cumulated response of neurons, which is also

used to derive the retinotopic boundaries. Both PCA and LDA are simple linear projections applied to the input responses. We again show an illustration of the LDA projection in Fig R7. The LDA projection (in Fig R7-B) can minimize the within-class variability and increase the between-class distance. This is because the clusters are distinct in the input x, y coordinate space (Fig R7-A). Thus, we argue that the LDA space can cluster neurons from different areas (Fig 9 in the text) using examples from the training data because the responses from different are are distinct in the input domain. We have used intra and inter-area correlations mainly as a tool to support this claim.

Our analysis does not speak further to what features of neuronal responses are responsible for the classification. The analysis pipeline does not use any explicit configuration of stimuli except averaging the response across trials. Hence, we have removed mention of "visual cortical areas have characteristic activity patterns" from the Abstract, and replaced it with "The results suggest that responses from visual cortical areas can be classified effectively using data-driven models."

Minor Comments

Comment 3.6:

Please indicate whether "resting state" for the wide field dataset is in the dark or with a gray screen?

Response:

Resting state responses for wide-field dataset, was collected in complete darkness, with the screen turned off. We now specify this clearly in the text (Section 1.1.1).

Comment 3.7:

The wide-field dataset methods say the mice are Ai93 and that all mice expressed GCaMP6f or GCaMP6s. Ai93 is exclusively GCaMP6f, so either the "or GCaMP6s" is a mistake, or another reporter line (possibly Ai94) was used?

Response:

Ai93 line was used for GCaMP6f mice only and Ai94 line was used for GCaMP6s mice. This information has been added to the text

Comment 3.8:

Mice expressing Emx1-IRES-Cre;Ai93 have been shown to have aberrant activity (Steinmetz et al 2017) – large cortex wide events. Do you think this could impact your results? In my mind, it seems unlikely for two reasons – one my recollection of the paper was that the aberrant activity was weaker in visual cortex than other areas; second, I would expect it to make pixels more similar to one another, so the fact that you are able to distinguish areas indicates that any aberrant activity likely isn't factoring into the clustering analysis. It might be worth adding a comment on this – but I leave that to the author's discretion.

Response:

As the reviewer pointed out, the aberrant activities are relatively rare and weak events. We do not believe this influences our results.

Comment 3.9:

Are you using the ΔF/F traces for the 2P dataset provided through the AllenSDK or the raw fluorescence? I would assume the former, but there are mentions of "raw neuronal activity" that make me wonder. This would be important to add to the methods.

Response:

Yes, we have always used dF/F traces. By "raw neuronal activity," we mean the responses given as input to the proposed pipeline. We have changed all references of "raw responses" to "input responses" or "responses given as input to the proposed model".

Comment 3.10

The citation for the Allen Brain Observatory should not be Lein et al. 2007, but should be de Vries, Lecoq, Buice et al 2020.

Response:

The citation has been fixed.

References

Garrett, M., Garrett, M., Nauhaus, I., Marshel, J. H., Marshel, J. H., & Callaway, E. M. (2014). Topography and Areal Organization of Mouse Visual Cortex. *The Journal of Neuroscience, 34*(37), 12587-12600. Retrieved 10 27, 2020, from https://ncbi.nlm.nih.gov/pmc/articles/pmc4160785

Juavinett, A. L., & Callaway, E. M. (2015). Pattern and component motion responses in mouse visual cortical areas. *Current Biology, 25*, 1759–1764.

Kobak, D., & Berens, P. (2019). The art of using t-SNE for single-cell transcriptomics. *Nature communications, 10*, 1-15.

Maaten, L. v., & Hinton, G. (2008). Visualizing Data Using t-SNE. *Journal of Machine Learning Research, 9*, 2579–2605. Retrieved 10 22, 2020, from http://jmlr.org/papers/volume9/vandermaaten08a/vandermaaten08a.pdf

Marshel, J. H., Garrett, M. E., Nauhaus, I., & Callaway, E. M. (2011). Functional specialization of seven mouse visual cortical areas. *Neuron, 72*, 1040–1054.

Smith, I. T., Townsend, L. B., Huh, R., Zhu, H., & Smith, S. L. (2017). Stream-dependent development of higher visual cortical areas. *Nature neuroscience, 20*, 200–208.

Wang, Q., Sporns, O., & Burkhalter, A. (2012). Network analysis of corticocortical connections reveals ventral and dorsal processing streams in mouse visual cortex. *Journal of Neuroscience, 32*, 4386–4399.

Yildirim, M., Hu, M., Le, N. M., Sugihara, H., So, P. T., & Sur, M. (2020). Quantitative third-harmonic generation imaging of mouse visual cortex areas reveals correlations between functional maps and structural substrates. *Biomedical Optics Express, 11*, 5650--5673.

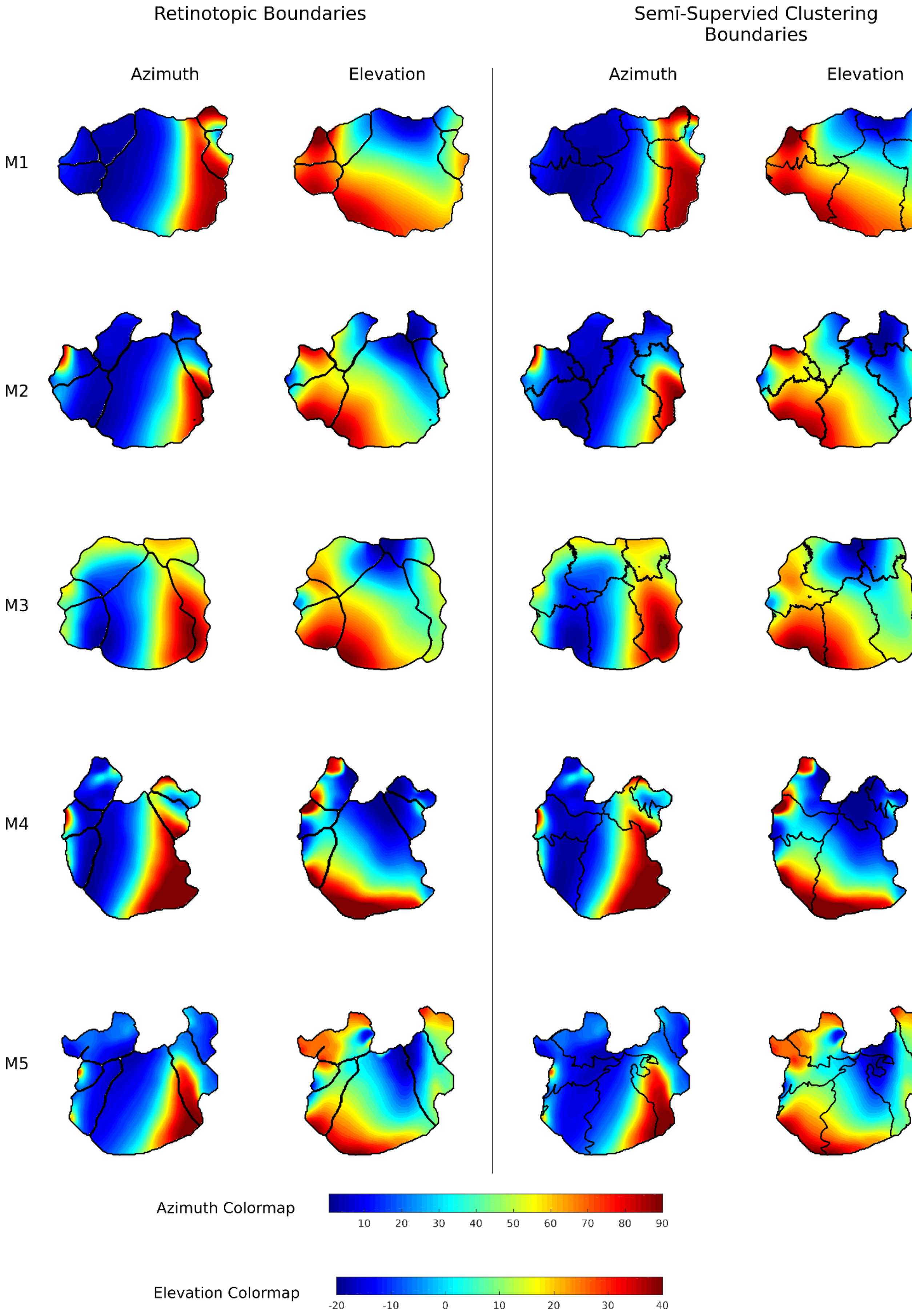

Fig R3 Comparison of semi-supervised clustering boundary to horizontal and vertical retintopy

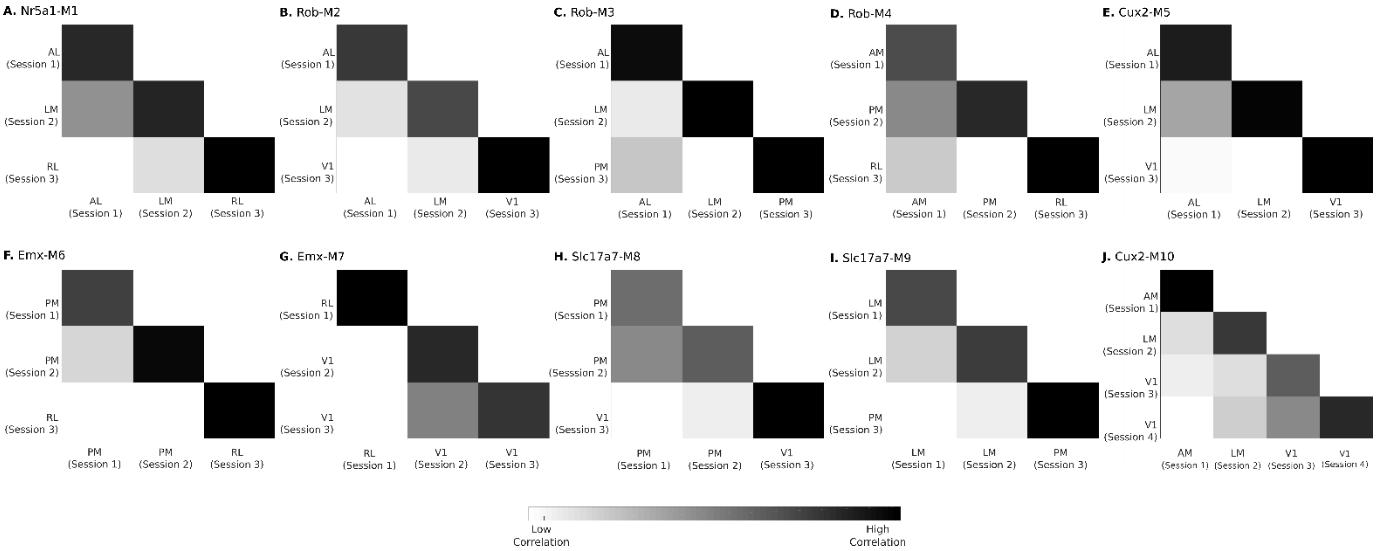

Fig R4 Intra-area and inter-area correlations computed from natural movies responses of individual mice from the two-photon dataset. In Figure 8, intra-area and inter-area correlations were computed at the Cre-line level with data pooled from different mice and sessions for the two-photon dataset. Here we present the inter and intra-area correlations for various individual mice from the dataset, which had responses recorded from three or more sessions.

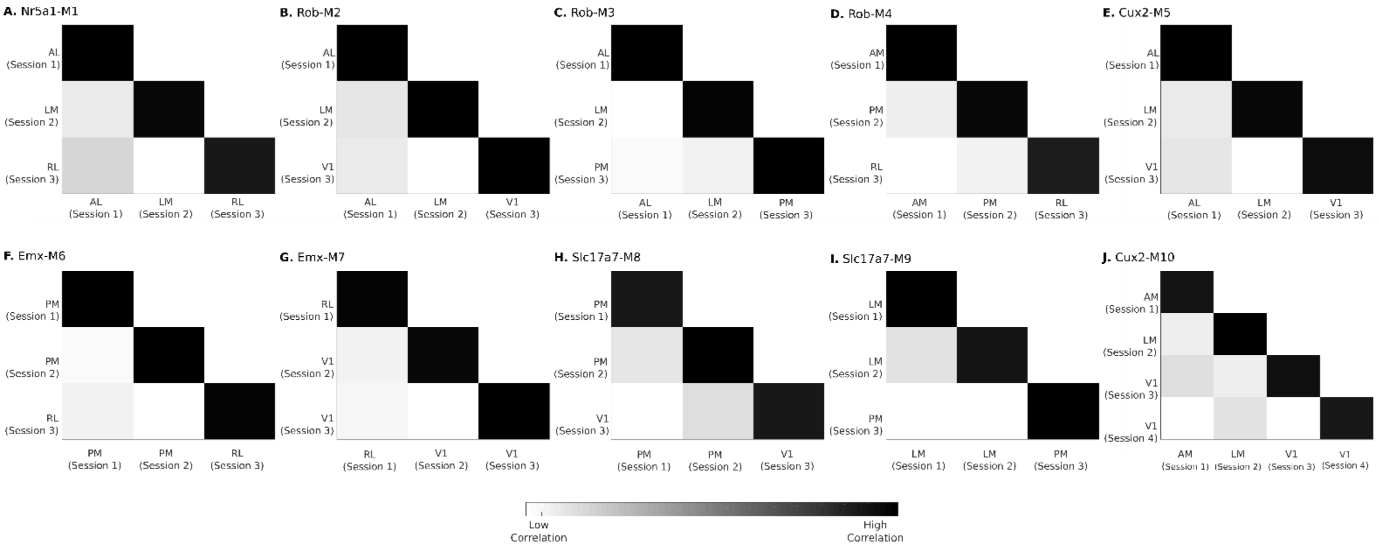

Fig R5 Intra-area and inter-area correlations computed from resting state responses of individual mice from the two-photon dataset. In Figure 8, intra-area and inter-area correlations were computed at the Cre-line level with data pooled from different mice and sessions for the two-photon dataset. Here we present the inter and intra-area correlations for various individual mice from the dataset, which had responses recorded from three or more sessions.

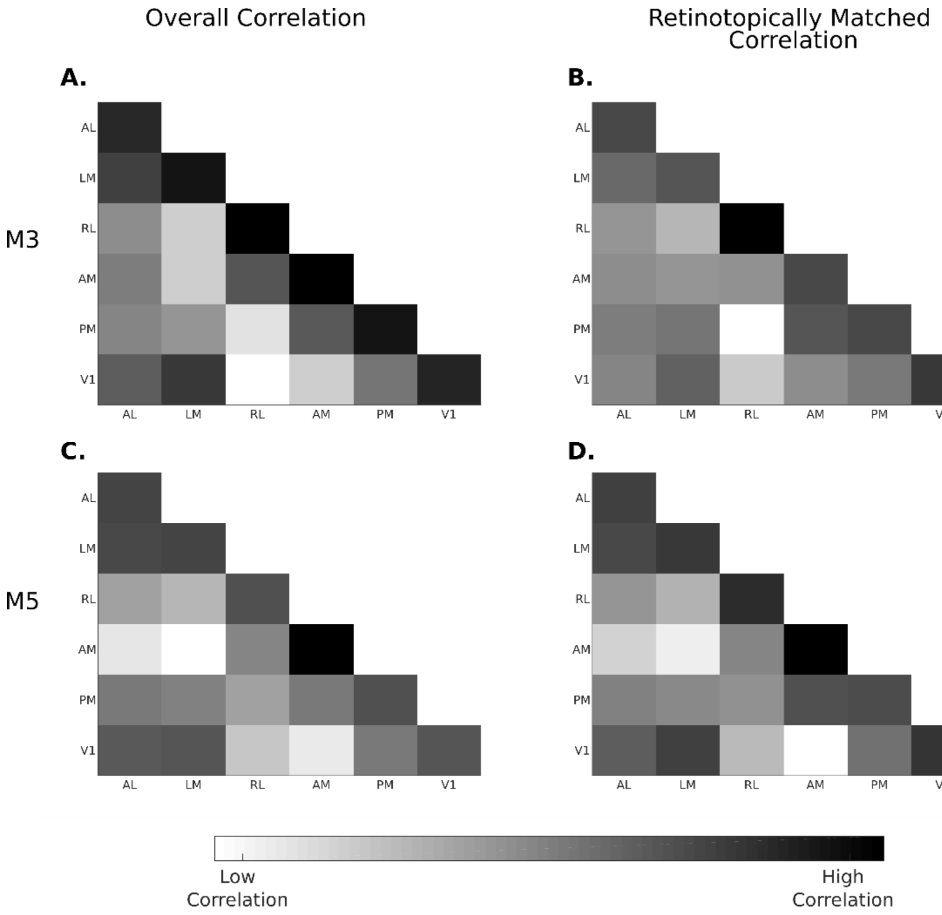

Fig R6 Comparison of intra and Inter-area correlation computed using all pixels and patches of pixels with the same retinotopic map. A, C) Intra and inter-area correlations computed using all pixels. **B, D)** Corresponding correlations computed using patches of pixels with approximately the same retinotopy from each area and averaged across the entire visual space. These data show that intra-area pixels are more correlated than inter-area pixels.

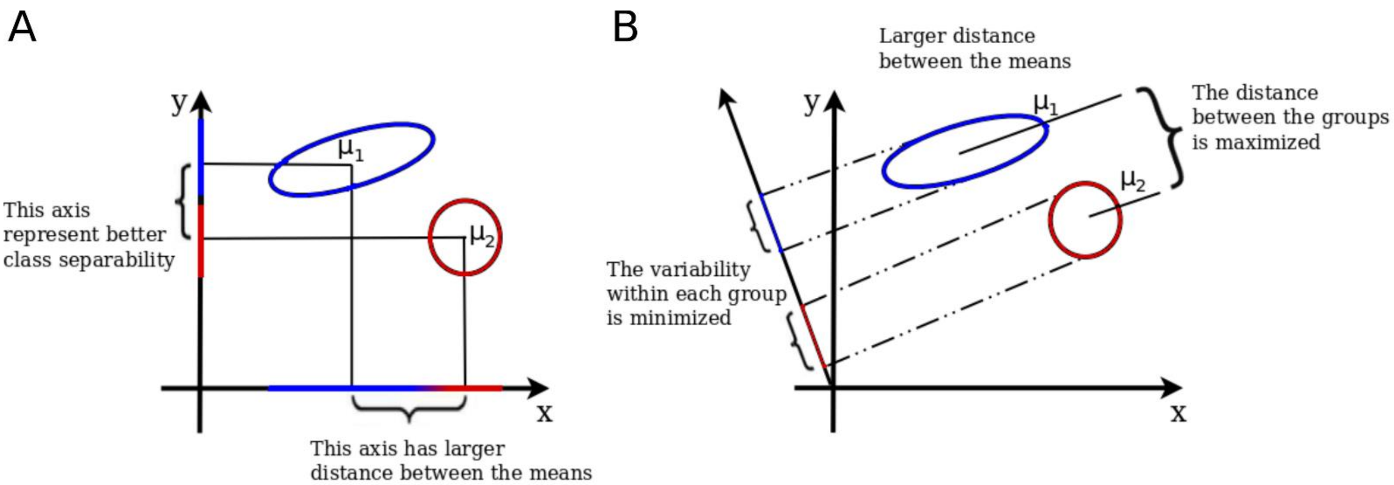

Fig R7 Illustration of LDA. A) Two clusters in x, y coordinate space. B) A possible LDA projection that minimizes within-class variability while increasing between class distance.
