# Supplementary material for: Functional parcellation of mouse visual cortex using statistical techniques reveals response-dependent clustering of cortical processing areas
Source: PLoS Comput Biol. 2021 Feb 4;17(2):e1008548. doi: 10.1371/journal.pcbi.1008548 (PMC7888605; doi:10.1371/journal.pcbi.1008548)
Supplement: Attachment — Submitted filename: review.docx [file pcbi.1008548.s011.docx]

PCOMPBIOL-D-20-00156R1

Functional Parcellation of Mouse Visual Cortex Using Statistical Techniques Reveals Response-Dependent Clustering of Cortical Processing Areas

I would thank the authors for the detailed answers they have provided to my questions. The paper’s much improved after their extensive revisions. The revised study adds an interesting new perspective to visual maps and to our understanding of mouse vision. With minor revisions, the updated manuscript would be suitable for publication in PLOS Comp Bio.

The authors have addressed most of my questions. I have two remaining.

(1) I remain convinced that the visual area borders are erroneous. The paper reports differences across identified visual areas so alignment to previously established borders is critical. I and the other reviewers all raised concerns about the maps and this problem needs to be resolved if this paper is to be meaningful.

The problem is less obvious now that there are no sign maps in the revised paper. The lack of sign maps, on which the border assignments are based, is a problem. I suggest the authors add field sign maps, with borders marked, to Fig R4 and add figure R4 to the paper.

The more fundamental problem remains that border locations are not where the field sign crosses zero. Borders at zero-crossing is basis field sign mapping. The authors’ claim that they have duplicated the field sign mapping approach of previous papers is inaccurate – this much is clear from their figures.

To illustrate the problem, below I’ve reproduced a panel from figure 1 of the original submission. Field sign = 0 is in the light blue/green color zone. The borders are generally not in the light blue-green zone and so at not at field sign = 0. Why is the border not at field sign = 0? The error needs to be found and resolved. The resulting shift in border positions may affect the results and conclusions.

(2) Where are the cluster-based borders and how do they relate to borders identified in previous papers?

Figure 9 indicates that some pixels from V1 cluster with pixels from LM, AL, PM and RL. I’ve indicated these pixels in the figure below, copied from the updated paper. Where are these pixels? Are they along the interface between V1 and adjacent areas? If they are, clustering predicts slightly different border locations that the field sign mapping procedure. Of course, field sign mapping predicts slightly different borders to architectonic methods (Zhuang et al., 2017). Do the cluster borders more closely match the field sign borders or architectonic borders, or does clustering identify a third set of borders? Answering these questions would link the paper directly to the existing literature.
